# A demographic theory of similarity-biased social learning

**DOI:** 10.1073/pnas.2606741123

**Published:** 2026-07-30

**Authors:** Alejandro Pérez Velilla, Paul E. Smaldino

**Affiliations:** ^a^ Department of Cognitive and Information Sciences, University of California, Merced, CA 95343; ^b^ Department of Human Behavior, Ecology and Culture, Max Planck Institute for Evolutionary Anthropology, Leipzig, Germany 04103; ^c^ Santa Fe Institute, Santa Fe, NM 87501; ^d^ Complexity Science Hub, Vienna 1030, Austria

**Keywords:** parochialism, cultural evolution, majority–minority dynamics, evolutionary model

## Abstract

We develop a demographic theory of similarity-biased social learning that formalizes our understanding of when and why individuals should preferentially copy others that look or act like them. We build an evolutionary model in which individuals can either learn on their own or copy others from a demonstrator pool that contains varying proportions of in-group and out-group members, and where group tags can be more or less informative about local knowledge. We find that where social learning becomes common, selection favors copying biases that track the direction of informational advantage—toward the group that tends to be better adapted to local conditions, including an antisimilarity bias when tags are negatively associated with local correctness (as may be the case for some immigrant communities). We also find conditions in which a similarity bias can stabilize social learning when such learners are already common, but not when they are rare, with implications for the role of group identities in cultural evolution. We discuss implications for understanding parochialism as risk aversion, majority–minority dynamics, the sociology of immigration, and the lasting impacts of colonialism.

## Significance

Humans adapt by learning from others, but risk acquiring maladaptive behaviors when learning from individuals facing different conditions. We present an evolutionary model exploring how individuals use social identity markers to select learning targets in diverse populations. Learners evolve biases that track reliable information sources: preferring similar individuals when in-group knowledge is adaptive, and dissimilar individuals when in-group behaviors are disadvantageous. Our framework demonstrates that this adaptive bias makes social learning viable even when it is costlier than individual learning. Additionally, we show that a minimum threshold of reliable cultural information is required for social learning to spread, providing a mathematical explanation for how demographic diversity drives population-level patterns of cultural segregation and assimilation.

Social learning is a central component of human adaptability (1–3). But social learning is not only about how much we copy; it is also about who we copy. We shall adopt the word “target” to indicate an individual being copied by a social learner. In large populations, learners encounter potential targets under conditions of demographic mixing, migration, and social stratification, and they routinely use perceptible cues of identity to decide whose information to trust. Formal models for understanding the evolution of social learning have typically ignored identity, focusing on when copying is favored in the face of learning costs and environmental uncertainty (4–8). Social-learning models that *have* accounted for similarity have focused on a narrow range of benefits, such as learning coordination norms (9) or improving the efficacy of conformity (10). Meanwhile, a parallel empirical and theoretical literature has documented that learners regularly filter potential targets using social categories and markers (11–17). What remains underdeveloped is a clear account of how demographic composition and tag informativeness jointly determine the extent to which individuals rely on social learning and the ways they use identity markers when they do.

Previous evolutionary simulations demonstrate that similarity-biased social learning is adaptive when individual learning is unreliable, indiscriminate copying is degraded by diversity, and identity signals are informative (18). This paper extends that work with an analytical model yielding closed-form predictions about evolutionary outcomes. This formalization clarifies the mechanism, generates general comparative statics, and derives results regarding antisimilarity bias and frequency-dependent selection.

The core idea is that identity markers matter for learning insofar as they predict transferability of information. Copying someone who is successful in their own circumstances is not necessarily helpful if the learner’s local payoffs are governed by a different behavioral optimum. This problem appears in spatially structured models when migration erodes the correlation between what worked for some individuals and what will work for those who learn from them (4–6).

An analogous problem arises within a single geographic setting whenever a population is diverse enough that groups face systematically different affordances, constraints, or locally appropriate behaviors. For example, the value of strategies for investment or communication may depend heavily on an individual’s socioeconomic, ethnic, or cohort identity. In such mixed settings, the value of a social tag is at least partly determined by its ability to partition the pool of potential targets into sources that are more or less likely to carry information or perform behaviors that are locally appropriate for the learner.

We consider a population in which individuals are marked by unambiguous group identity tags—we do not consider ambiguous or covert markers of identity here (19). We treat group tags as imperfect informational cues and build a demographic theory around two ingredients. First, a learner samples from a demonstrator pool in which some individuals share the learner’s tag and others do not. Second, tags vary in informativeness: Sometimes tag identity predicts the local correctness of behavior, sometimes it is uninformative, and sometimes it actually predicts that a target’s behavior is unlikely to be locally correct. Learners can respond by evolving a continuous filtering trait that shifts attention toward either same-tag or different-tag targets, potentially paying a cost for stronger filtering. This setup compresses a wide range of substantive scenarios—migration, residential mixing, minority/majority structure, interaction across age cohorts, and other forms of heterogeneous social exposure—into a small number of interpretable demographic and informational parameters. Age structure supplies an example: Members of an age cohort are genuinely more likely to face the challenges—and thus to carry the locally appropriate behaviors—of their shared life stage. The cohort tag is typically unambiguous, and copying within it reflects the adaptiveness of the behavior itself rather than its prestige. The same parameters can also describe settings involving migration and majority/minority relationships, where the informational mechanism is typically entangled with status and power; we take up both kinds of cases in *Discussion*.

Our model yields four key predictions. First, tag-based biases track the group with the highest probability of executing locally adaptive behaviors. Second, this mechanism generates antisimilarity bias when a group’s markers correlate with maladaptive outcomes. Third, smaller populations experiencing higher sampling variance evolve stronger biases. Fourth, when unbiased social learning is disadvantageous with respect to individual learning but optimally biased social learning is not, cultural accumulation presents an invasion barrier: Social learning requires surpassing a threshold frequency, below which populations revert to individual learning, and above which positive feedback drives populations toward social learning.

We first present the general model and characterize its evolutionary outcomes, emphasizing how demographic composition and tag informativeness govern both the basin structure for social learning and the long-run tradeoff between widening the field of possible targets and narrowing that field by filtering out those who do not share one’s social tags. We then present the main comparative statics and characterize the long-run evolutionary regimes of the model.

## The Model

We consider a large population in which individuals can observe one another freely, but belong to distinct socioecological niches for which different behaviors are locally adaptive. An individual’s task is to acquire, through learning, a behavior that is optimal for their particular niche. Optimal behaviors are assumed to confer a high fitness payoff, while all other behaviors are suboptimal and confer a lower fitness payoff. Individuals can learn either through costly individual learning, or through social learning by copying the behavior of a target individual in the previous generation. Individuals are marked with an observable tag that, from the perspective of a learner, partitions potential targets into either in-group (sharing their tag) or out-group (not sharing their tag). Learners may use this tag to bias their choice of target toward either similar or dissimilar individuals. We focus on the coevolution of traits controlling social learning and similarity bias.

An individual uses social learning with probability q, and individual learning otherwise. Individual learning incurs a fixed cost k and succeeds in acquiring the optimal behavior with probability s. If an individual learner fails to acquire the optimal behavior, they incur an additional cost β. For simplicity, we set the gross payoff for acquiring the optimal behavior to 1. Thus, the expected payoff to an individual learner is

[1] $$\begin{array}{c} V_{\mathrm{IL}}=1-\beta \left(1-s\right)-k. \end{array}$$

**Fig. 1.**
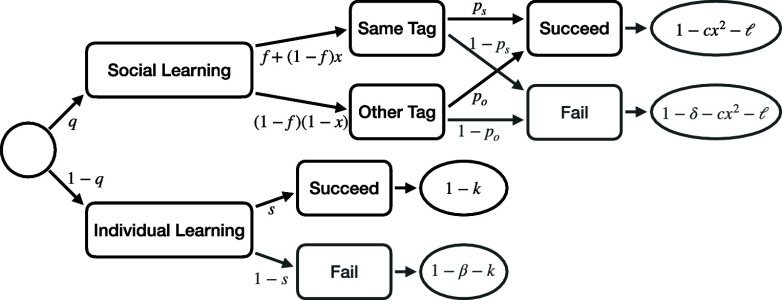
Model diagram. Flowchart of decisions and payoffs from the perspective of an individual agent. The chart assumes x≥0.

The success of a social learner depends on the frequency of individuals using the optimal behavior for the learner’s niche during the current time period t, $Q_{t}$. A social learner must also make the additional choice of whom to copy: someone with their own tag or another tag. For a given learner, let f be the frequency of similarly tagged individuals in their local pool of potential targets. Note that payoffs are normalized so that the baseline outcome yields 1: The penalty β is subtracted only when individual learning fails, and the fixed cost k is paid regardless of outcome. A successful individual learner therefore earns 1−k and an unsuccessful one earns 1−β−k, consistent with Fig. 1. An individual expresses a similarity bias, x∈[−1,1], so that x>0 indicates a bias for similarity and x<0 indicates a bias for learning from dissimilar targets. The probability that a learner selects a target with their own tag is a function of both f and x:

[2] $$\begin{array}{c} \mathrm{Pr}\left(\text{own tag}|x,f\right)=\left\{\begin{array}{cc} f+(1-f)\;x, & x\geq 0,\\ f+f\;x, & x<0. \end{array}\right. \end{array}$$

This formulation ensures that sampling probabilities remain feasible ([0,1]) for any bias x whenever the pool contains both types (0<f<1); we write the conditioning on f explicitly to mark this dependence on pool composition. The endpoint cases f=0 and f=1, in which one group is wholly absent, are treated only as limits, and all derivations below are stated for 0<f<1.

We assume that social learning carries a fixed cost, ℓ<k, and that a bias for or against similar targets carries an additional cost that scales with the magnitude of the bias. A symmetric cost function that satisfies these constraints is $cx^{2}$. We treat this term as a reduced-form representation of the costs of stronger filtering—cognitive effort, attentional load, and the opportunity cost of a more restricted sampling pool—rather than as a commitment to any single psychological mechanism. The quadratic form is the simplest symmetric specification that yields interior optima; linear or higher-order costs would shift quantitative predictions but leave the qualitative regime structure intact. A social learner that fails to acquire the optimal behavior incurs an additional mismatch penalty δ. We allow this to differ from the cost of failed individual learning to reflect the possibility that learning from a different type of person may be better (or worse) than the penalty for failed individual learning. The decisions involved in learning behaviors and the parameters governing their probabilities and outcomes are illustrated in Fig. 1. A list of all model parameters is given in Table 1.

**Table 1.** Model parameters

Symbol	Definition	Range
q	Probability of social learning (SL)	[0,1]
x	Similarity bias	[−1,1]
s	Individual-learning (IL) accuracy	$\left[\frac{1}{2},1\right]$
k	Fixed cost of IL	[0,+∞]
β	Penalty if IL is wrong	[0,1]
δ	Penalty if SL is wrong	[0,1]
c	Marginal cost of bias	[0,+∞]
ℓ	Marginal cost of SL	[0,+∞)
f	Frequency of own-tag in pool	[0,1]
ρ	Tag reliability	[−1,1]
γ	Crowding cost of SL	[0,+∞)
$N_{e}$	Effective population size	$N_{>0}$

Payoffs are scaled so that niche-appropriate behavior yields 1. The accuracy s and tag reliability ρ are treated as independent primitives; the ranges listed are individual admissible ranges, not a joint restriction. Several displayed expressions are interior formulas requiring q<1, c>0, and 0<f<1; the boundary cases (q→1, c→0, and f∈{0,1}) are handled separately where they arise. The feasibility extension makes the cultural recursion well-defined for every pair (s,ρ), so no joint restriction on the two is imposed.

We can define tag reliability, ρ, as the marginal probability of acquiring the optimal behavior by copying a similar target:

[3] $$\begin{array}{c} \rho \equiv p_{s,t}-p_{o,t} \end{array}$$

When ρ>0, the tag is a reliable marker of local adaptation; when ρ<0, the tag is misleading (anticorrelated with correctness). Indeed, we show in *SI Appendix* that the tag reliability ρ is exactly equivalent to the regression coefficient of behavioral correctness on tag identity. Using substitutions derived from Bayes’ Theorem (derivation in *SI Appendix*), we can rewrite the conditional accuracies in terms of the sample pool quality and the tag reliability:

[4] $$\begin{array}{c} p_{s,t}=Q_{t}+\left(1-f\right)\rho ,\;p_{o,t}=Q_{t}-f\rho . \end{array}$$

It follows that the probability of successfully acquiring the optimal behavior for a social learner, $M_{t}\left(x\right)$, is

[5] $$\begin{array}{c} M_{t}\left(x\right)=\;\mathrm{Pr}\left(\text{own tag}|x,f\right)\;p_{s,t}+\left(1-\mathrm{Pr}(\text{own tag}|x,f)\right)p_{o,t} \end{array}$$

Substituting the equivalences from Eq. **4** yields the following compact expression:

[6] $$\begin{array}{c} M_{t}\left(x\right)=\left\{\begin{array}{cc} Q_{t}+\left(1-f\right)\rho \;x, & x\geq 0,\\ Q_{t}+f\rho \;x, & x<0. \end{array}\right. \end{array}$$

This equation summarizes the mechanics of biased social learning: The probability of acquiring correct behavior is the baseline pool quality $Q_{t}$, shifted by the product of the tag’s information (ρ) and the learner’s bias (x), scaled by the demographic leverage available (1−f or f).

### Steady-State Information Quality.

In a population in which the nature of social learners (q,x) is fixed, the overall quality of social information will evolve across time periods. In each time period, the adult pool is refreshed by a mixture of individual learners, who introduce new information with accuracy s, and social learners, who propagate existing information with accuracy $M_{t}\left(x\right)$. The frequency of correct behavior in the next time period, from the perspective of a social learner’s local pool, is given by the following recursion:

[7] $$\begin{array}{c} Q_{t+1}=\left(1-q\right)\;s\;+\;q\;M_{t}\left(x\right). \end{array}$$

Substituting the biased copying probability from Eq. **6** isolates the specific contribution of similarity bias:

[8] $$\begin{array}{c} Q_{t+1}=\left(1-q\right)\;s\;+\;q\left(Q_{t}\;+\;\rho \;x\;\vartheta \left(x\right)\right), \end{array}$$

where

[9] $$\begin{array}{c} \vartheta \left(x\right)=\left\{\begin{array}{cc} 1-f, & x>0,\\ f, & x<0,\\ 0, & x=0, \end{array}\right. \end{array}$$

captures the demographic leverage available for filtering. This is essentially the extent to which the population contains individuals that a biased social learner can exclude.

Assuming that each generation is composed of many time periods representing population learning and cultural transmission episodes, the quality of social information will reach a steady state when $Q_{t+1}=Q_{t}=Q^{*}$. This is the fixed point for cultural accuracy in a given local niche:

[10] $$\begin{array}{c} Q^{*}\left(q,x\right)={\text{clip}}_{\left[0,1\right]}\left[s+\left(\frac{q}{1-q}\right)\rho \;x\;\vartheta \left(x\right)\right]. \end{array}$$

The term $\frac{q}{1-q}$ is the cultural multiplier, which represents the expected number of times a piece of information is socially transmitted before being lost. When social learning is common (q→1), this memory factor grows large, amplifying the effect of even weak bias x or weak tag reliability ρ on the standing crop of knowledge. Conversely, if cues are uninformative (ρ=0), social learning is rare (q≈0), or learners are unbiased (x=0), the pool reverts to the baseline accuracy of individual learning, $Q^{*}=s$.

While Eq. **10** describes the pressure to accumulate accuracy, the system is physically constrained by demographic mixing. Because the conditional probabilities $p_{s,t}$ and $p_{o,t}$ must remain in [0,1], the steady-state accuracy cannot exceed the bounds imposed by the tag-correctness correlation. From Eq. **4**, the admissible interval for Q is $\left[Q_{\min},Q_{\max}\right]$, where:

[11] $$\begin{array}{c} Q_{\min}\left(\rho ,f\right)=\;\max\left\{f\rho ,-\left(1-f\right)\rho \right\}, \end{array}$$

[12] $$\begin{array}{c} Q_{\max}\left(\rho ,f\right)=\;\min\left\{1+f\rho ,1-\left(1-f\right)\rho \right\}. \end{array}$$

The realized cultural steady state is therefore the interior solution clipped to these feasibility bounds:

[13] $$\begin{array}{c} Q\left(q,x\right)=\;\min\{\max\{\;Q^{*}\left(q,x\right),\;Q_{\min}\;\},\;Q_{\max}\;\}. \end{array}$$

This clipped expression should be read as a feasibility projection: When the unconstrained recursion would produce a value outside the admissible interval $\left[Q_{\min},Q_{\max}\right]$, the realized pool is held at the nearest feasible boundary by the demographic constraints on $p_{s,t}$ and $p_{o,t}$; payoffs are then evaluated at this projected value. The projection coincides with the recursion’s fixed point whenever $Q^{*}\left(q,x\right)\in \left[Q_{\min},Q_{\max}\right]$ and otherwise records the closest accuracy the population can actually attain.

The lower bound also has a natural interpretation that extends the recursion to the full interval [0,1]. Because ρ is a difference of conditional probabilities of correctness, an empty or near-empty pool cannot support an informative tag; we accordingly treat ρ as the environmental value of the correlation *when it is feasible*, with |ρ|=0 on $\left[0,Q_{\min}\right)$, so the cultural recursion reduces to the unbiased form there. The upper bound is realized by clipping at $Q_{\max}$. This extension is what lets us treat s and ρ as independent environmental primitives rather than imposing a joint admissibility restriction: Because ρ is inert wherever the pool lies below $Q_{\min}\left(\rho ,f\right)$, the recursion is well defined for any pair (s,ρ), including pairs for which the unbiased fixed point $Q^{*}=s$ would itself fall outside $\left[Q_{\min},Q_{\max}\right]$—the tag simply carries no realized information at the resident pool, and no combination of primitives needs to be excluded a priori. The lower bound matters dynamically only when tags are strongly informative and demographic shares are sharply asymmetric, so that $Q_{\min}$ approaches or exceeds s; in the adaptive regimes we analyze below the binding constraint is the upper bound $Q_{\max}$, whose saturation dynamics we take up in the analysis of saturation and crowding. Throughout the adaptive regimes analyzed below, payoffs are evaluated at this $Q_{\max}$-constrained steady state.

### Evolutionary Dynamics.

We can now derive selection gradients for our two evolving traits, q and x, and assess their evolution across generations. We consider a resident population with strategy (q,x) that determines the steady-state accuracy of the cultural pool, Q(q,x). A rare mutant individual with strategy $\left(q^{\prime },x^{\prime }\right)$ then competes against this background.

The expected payoff for individual learning is constant regardless of the cultural environment (Eq. **1**). To derive the expected payoff for an invading social learner, we note that their success depends on both the accuracy of the resident cultural pool, Q, and their own filtering bias $x^{\prime }$. Let $M(Q,x^{\prime })$ denote the probability that a social learner acquires the correct behavior:

[14] $$\begin{array}{c} M\left(Q,x^{\prime }\right)=Q+\rho \;\vartheta \left(x^{\prime }\right)x^{\prime }. \end{array}$$

The expected payoff for a mutant social learner is therefore:

[15] $$\begin{array}{c} V_{\mathrm{SL}}\left(q^{\prime },x^{\prime }|q,x\right)=1-\delta [1-M\left(Q\left(q,x\right),x^{\prime }\right)]-c{\left(x^{\prime }\right)}^{2}-\ell . \end{array}$$

The fitness of the invading mutant, $W_{\mathrm{mut}}$, is the weighted average of these payoffs:

[16] $$\begin{array}{c} W_{\mathrm{mut}}\left(q^{\prime },x^{\prime }|q,x\right)=\left(1-q^{\prime }\right)V_{\mathrm{IL}}+q^{\prime }V_{\mathrm{SL}}\left(q^{\prime },x^{\prime }|q,x\right). \end{array}$$

Evolution proceeds along the selection gradients defined by the partial derivatives with respect to the mutant traits, evaluated at the resident state $\left(q^{\prime }=q,x^{\prime }=x\right)$. As is standard in this literature, the dynamics treat strategy frequencies as changing in proportion to payoff advantage. This admits both a biological reading (strategies inherited under a replicator dynamic) and a behavioral one (agents preferentially adopting higher-payoff learning rules); we do not distinguish between them, requiring only that frequencies move up the payoff gradient. We also treat the learning decision as resolved once per generation, a common simplification we return to in *Discussion*.

The selection gradient on bias, $w_{x}$, describes the marginal private benefit of filtering. Because a mutant individual’s bias affects only their own success and not the pool they sample from, the gradient depends only on the immediate informational gain:

[17] $$\begin{array}{c} w_{x}=\frac{\partial W_{\mathrm{mut}}}{\partial x^{\prime }}{|}_{x^{\prime }=x}=q\left[\delta \rho \vartheta (x)-2cx\right]. \end{array}$$

The factor q reflects that bias only matters when the mutant actually uses social learning. Inside the brackets, the first term is the marginal informational gain from increasing bias—positive when the bias points in the direction of tag reliability, negative otherwise. The second term is the marginal cost 2cx of the quadratic bias penalty.

Setting $w_{x}=0$ yields an optimal bias

[18] $$\begin{array}{c} x^{*}=\frac{\delta \;\rho \;\hat{\vartheta }}{2c} \end{array}$$

that is independent of the frequency of social learning (q). Here $\hat{\vartheta }\equiv \vartheta \left(x^{*}\right)$, so $\hat{\vartheta }=1-f$ when ρ>0, $\hat{\vartheta }=f$ when ρ<0, and $\hat{\vartheta }=0$ when ρ=0. Notice that bias is predicted to favor similarly tagged targets when tags are correlated with optimal behavior, to favor dissimilarly tagged targets when tags are anticorrelated with optimal behavior, and to ignore tags completely when they are uninformative.

The selection gradient on social learning, $w_{q}$, is the payoff difference between social and individual learning given the current cultural environment:

[19] $$\begin{array}{c} w_{q}=\frac{\partial W_{\mathrm{mut}}}{\partial q^{\prime }}{|}_{q^{\prime }=q}=V_{\mathrm{SL}}-V_{\mathrm{IL}}. \end{array}$$

Substituting our equation (Eq. **10**) for the steady-state resident pool accuracy $Q^{*}\left(q,x\right)$, we obtain:

[20] $$\begin{array}{c} w_{q}=\left(\beta -\delta \right)\left(1-s\right)+k-\ell +\delta \left(\frac{\rho x\vartheta (x)}{1-q}\right)-cx^{2}. \end{array}$$

It is helpful here to name two grouped terms that summarize the incentives for social learning. First, let L denote the baseline disadvantage of unbiased social learning:

[21] $$\begin{array}{c} L\equiv \ell -(k+(\beta -\delta )(1-s)) \end{array}$$

It is the net fitness cost of copying a random individual relative to learning individually. When L>0, unbiased social learning is the more expensive strategy; when L<0—if individual learning is sufficiently costly or error-prone—unbiased copying is already favored, and bias serves only to amplify the advantage.

Second, let B denote the bias-induced gain: the net payoff improvement that the optimal bias $x^{*}$ provides over unbiased copying (full derivation in *SI Appendix*). When the optimum is interior—when the unconstrained value $\delta \rho \hat{\vartheta }/\left(2c\right)$ lies within [−1,1], so the clip introduced in the Results does not bind—this gain takes the closed form

[22] $$\begin{array}{c} B\equiv \frac{{\left(\delta \;\rho \;\hat{\vartheta }\right)}^{2}}{4c}\geq 0. \end{array}$$

B collapses to zero if any of mismatch cost, tag reliability, or demographic leverage vanishes: Bias helps only when errors are costly, the tag is informative, and there is a group to filter out. The complementary boundary regime, in which $|x^{*}|=1$ and Eq. **22** no longer applies, is treated in the Results; until then expressions are stated for the interior regime.

With these definitions, the selection gradient on social learning simplifies, in the interior regime, to

[23] $$\begin{array}{c} w_{q}\left(q\right)=\frac{2B}{1-q}-\left(L+B\right)\;\text{(interior).} \end{array}$$

The term $\frac{1}{1-q}$ captures the frequency-dependent benefit of copying. As social learning becomes common, the resident pool accumulates accuracy, increasing the incentive for others to adopt social learning. B plays a double role in this expression: In the first term, it scales the benefits of biased copying, and in the second term, it represents the added costs of higher bias.

### Finite Populations and Risk.

For finite populations of effective size $N_{e}$ without aggregate between-generation variation, we can follow ref. 20 in assuming that selection on a strategy z will act to maximize the following fitness expression:^*^

[24] $$\begin{array}{c} W\left(z\right)=V_{z}-\frac{\sigma^{2}\left(z\right)}{N_{e}}, \end{array}$$

where $V_{z}$ is the mean fitness of strategy z and $\sigma^{2}\left(z\right)$ is the within-generation variance in the fitness of individuals using strategy z. In infinite populations, the variance penalty in the right hand side becomes negligible, but small populations can face significant variance penalties. Our strategy comparison must then include the within-generation variance terms. The variance in fitness of a pure IL agent is given by

[25] $$\begin{array}{c} \sigma_{\text{IL}}^{2}=\beta^{2}s\left(1-s\right), \end{array}$$

while the variance for a pure SL agent is

[26] $$\begin{array}{c} \sigma_{\text{SL}}^{2}=\delta^{2}M\left(1-M\right). \end{array}$$

Let $M^{*}\equiv M\left(Q,x^{*}\right)$ be the maximum accuracy of a social learning. Social learning will have an evolutionary advantage over individual learning in small populations when

[27] $$\begin{array}{c} V_{\text{SL}}\left(Q,x^{*}\right)-\frac{\delta^{2}}{N_{e}}M^{*}\left(1-M^{*}\right)>V_{\text{IL}}-\frac{\beta^{2}}{N_{e}}s\left(1-s\right), \end{array}$$

so that the selection gradient on social learning becomes

[28] $$\begin{array}{c} w_{q}\left(Q,x^{*};N_{e}\right)=0⟺V_{\text{SL}}\left(Q,x^{*}\right)-V_{\text{IL}}=\frac{\sigma_{\text{SL}}^{2}-\sigma_{\text{IL}}^{2}}{N_{e}}. \end{array}$$

With our model thus defined and equations for its dynamics derived, we present the result of our analyses and their consequences for the evolution of similarity-biased social learning.

## Results

We first analyze the evolution of the population when the cultural pool is below the accuracy ceiling ($Q<Q_{\max}$). In this regime, individuals maximize their invasion fitness by simultaneously optimizing their propensity for social learning (q) and their strength of similarity bias (x).

### Optimal Bias Scales with Tag Reliability, Mismatch Costs, and Demographic Leverage.

In well-mixed populations, the optimal similarity bias, $x^{*}$, is determined solely by the private trade-off between the informational value of the tag and the cost of filtering. Setting $w_{x}=0$ yields an optimal bias that is independent of the current frequency of social learning:

[29] $$\begin{array}{c} x^{*}\left(\rho ,f\right)={\text{clip}}_{\left[-1,1\right]}\left(\frac{\delta \;\rho }{2c}\;\left\{\begin{array}{cc} 1-f, & \rho >0,\\ f, & \rho <0,\\ 0, & \rho =0, \end{array}\right.\right). \end{array}$$

This expression is the optimal bias conditional on social learning being expressed. Because the selection gradient on bias in Eq. **17** is directly proportional to q, bias is selectively neutral in a population near q=0: With no social learners, there is no filtering for selection to act on. Eq. **29** should therefore be read as the bias a social learner would adopt once copying is sufficiently common, not as a standalone prediction about populations of pure individual learners.

**Fig. 2.**
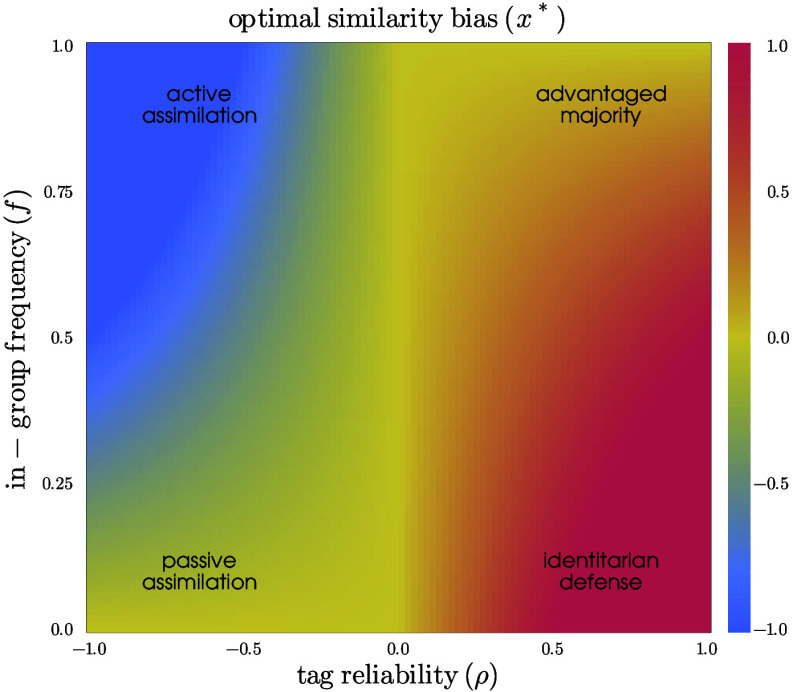
Regimes of optimal similarity bias ($x^{*}$). The heatmap shows the evolved strength of bias as a function of marker reliability (ρ) and in-group encounter rate (f). Blue regions indicate positive similarity bias, where learners preferentially copy in-group members; red regions indicate antisimilarity bias, where learners preferentially copy out-group members. The asymmetry arises because filtering leverage depends on the encounter rate with the “misleading” group. Plotted for δ=0.8 and c=0.2.

Subject to that proviso, similarity bias at equilibrium scales linearly with tag reliability ρ and the mistake penalty δ, increases with the available filtering leverage (1−f when ρ>0 and f when ρ<0), and decreases with the bias cost c. Fig. 2 shows how the space of optimal bias is divided as a function of tag reliability ρ and in-group encounter rate f. We identify four regimes of interest: i) the identitarian defense regime, where rare encounters with in-group demonstrators (f→0) coupled with positive tag reliability (ρ>0) yield strong similarity bias; ii) the active assimilation regime, where common encounters with in-group demonstrators (f→1) and negative tag reliability (ρ<0) yield strong antisimilarity bias; iii) the advantaged majority regime, where common encounters with in-group demonstrators (f→1) under a positive tag reliability (ρ>0) yield weakening incentives for similarity bias; and iv) the passive assimilation regime where a low encounter rate with in-group demonstrators (f→0) keeps antisimilarity bias low despite a negative tag reliability (ρ<0). We explore the significance of these regimes for real-world cultural dynamics in *Discussion*.

### The Boundary Regime: Saturated Bias.

When tag reliability is high, demographic leverage is large, or filtering is cheap, the unconstrained optimum $\delta \rho \hat{\vartheta }/\left(2c\right)$ can exceed 1 in magnitude and the clip in Eq. **29** binds. The optimal bias then saturates at $|x^{*}|=1$: The learner copies purely within, or purely outside, their own tag, with no intermediate tuning available. In this *boundary regime* the informational gain becomes the full linear term $\delta \rho \;x^{*}\vartheta \left(x^{*}\right)=\delta \left|\rho \right|\hat{\vartheta }$, no longer discounted by the quadratic cost structure, and the realized bias cost is $c{\left(x^{*}\right)}^{2}=c$. Defining the *gross informational gain*

[30] $$\begin{array}{c} G\equiv \delta \;|\rho |\;\hat{\vartheta }, \end{array}$$

the selection gradient on social learning in the boundary regime is

[31] $$\begin{array}{c} w_{q}\left(q\right)=\frac{G}{1-q}-\left(L+c\right)\;\text{(boundary).} \end{array}$$

The two regimes are structurally parallel, but differ in how gain and cost scale: In the interior regime both scale jointly with ${\left(\delta \rho \hat{\vartheta }\right)}^{2}/c$, while in the boundary regime the gain G scales linearly with $\delta |\rho |\hat{\vartheta }$ and the bias cost c is independent of tag informativeness, because the saturated filter produces additional gain from tag reliability at no additional cost. The substitutions 2B→G and L+B→L+c translate every interior-regime result below into its boundary-regime counterpart.

### The Evolution of Tag-Biased Social Learning Exhibits Frequency Dependence.

Selection on bias switches on only once social learning is common enough to be worth filtering, which requires the population to first cross an invasion threshold in q. This frequency dependence can generate bistability. If the baseline disadvantage of social learning over individual learning is positive (L>0) but filtering is beneficial (B>0) and social learning is costly when rare ($w_{q\to 0}=B-L<0$), the population must cross a critical threshold frequency of social learning, $q^{†}$, to sustain cumulative culture:

[32] $$\begin{array}{c} q^{†}=\left\{\begin{array}{cc} \frac{L-B}{L+B}, & L>B,\\ 0, & L\leq B. \end{array}\right. \end{array}$$

Below this threshold, the cultural pool is too degraded to justify the costs of copying, and the population shifts to rely entirely on individual learning (q→0). Above the threshold, the self-reinforcing quality of the target pool drives the frequency of social learning upward to fixation. The same threshold applies in the boundary regime under the substitution 2B→G, L+B→L+c: A population in the boundary regime must cross $q^{†}=\left(L+c-G\right)/\left(L+c\right)$ when G<L+c, and social learning invades unconditionally ($q^{†}=0$) otherwise.

**Fig. 3.**
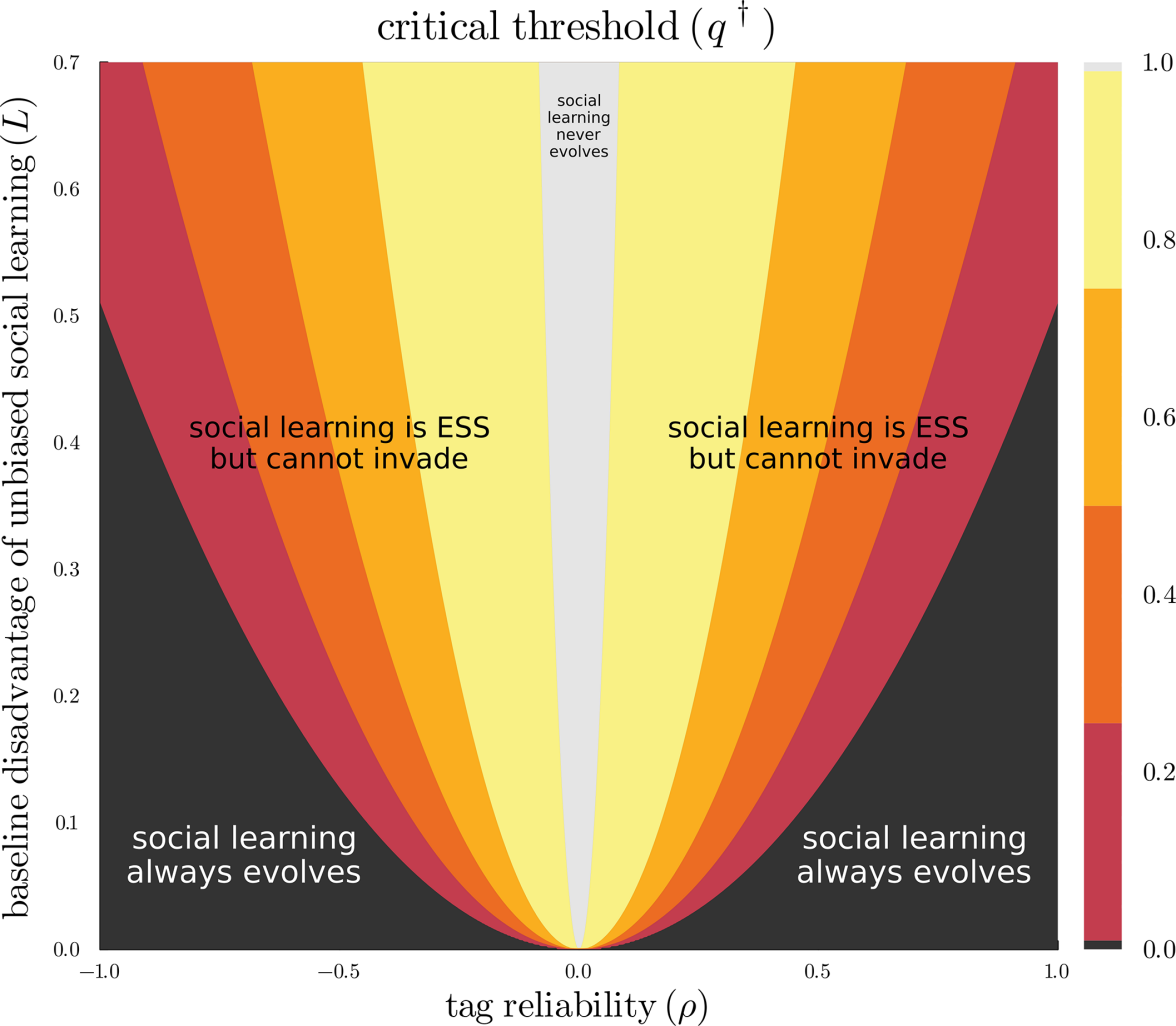
The catalytic role of tag reliability. The heatmap displays the critical threshold frequency ($q^{†}$) required for social learning to invade the population, plotted against marker reliability (ρ) and the baseline disadvantage of unbiased social learning (L). Darker regions indicate low thresholds where social learning can evolve from rare; brighter regions indicate high thresholds where the barrier to entry is insurmountable. The narrowing “U-shape” shows how reliable social markers act as a catalyst for cultural emergence: As the informational value of tags increases (|ρ|→1), the population can sustain culture even at significantly higher costs. Plotted for $\frac{\delta \hat{\vartheta }}{2c}=0.5$.

The critical social-learning frequency is fixed at $q^{†}=0$ whenever the benefit of optimal bias equals or exceeds the disadvantage of unbiased social learning relative to individual learning (B≥L). On the contrary, if L>B, $q^{†}$ increases asymptotically to 1 as L increases. More specifically, $q^{†}$ increases with anything that raises L: increases in the direct cost of social learning (ℓ) or decreases in the costs of individual learning (k, β). It decreases with anything that raises B: increases in |ρ|, in $\hat{\vartheta }$, or decreases in c. The mismatch penalty δ enters through both channels: It raises L (failed copies are more costly), which pushes $q^{†}$ upward, and it raises B (the value of a successful bias-conditioned copy is larger), which pushes $q^{†}$ downward. The net effect of δ on $q^{†}$ is therefore not monotone; the two channels reappear separately in the discussion of the interior equilibrium $q^{*}$ below. The threshold also increases with the accuracy of individual learning (s), though only when δ<β; for δ>β, increasing s decreases $q^{†}$, and s has no effect when δ=β. These factors represent the “break-even” point: If individual learning is cheap, accurate, and carries low penalties for errors, or if filtering out bad information is expensive, the population requires a larger existing culture of useful social information to justify the switch. Reliable social markers and opaque environments therefore act as catalysts, lowering the demographic barrier required to kickstart the cumulative cultural process (Fig. 3).

### Saturation, Crowding, and the Return of Rogers’ Paradox.

Consider now a population in which the conditions support the evolution of social learning. As the frequency of social learning (q) increases, the accuracy of the cultural pool eventually hits the demographic ceiling ($Q_{\max}$). The saturation analysis below applies in the regime where the cultural ceiling lies at or above the individual-learning baseline, $Q_{\max}\left(\rho ,f\right)\geq s$, so that there is room for biased copying to accumulate accuracy above IL accuracy (s).^†^ This occurs when the cultural multiplier is strong enough that the pool becomes as accurate as the tag-correctness correlation allows, which occurs at

[33] $$\begin{array}{c} q_{\mathrm{sat}}=\frac{Q_{\max}-s}{\left(Q_{\max}-s\right)+\rho \;\hat{\vartheta }\;x^{*}}, \end{array}$$

where $\hat{\vartheta }\equiv \vartheta \left(x^{*}\right)$ represents the demographic leverage available at the optimal bias. This expression is regime-neutral: The product $\rho \hat{\vartheta }x^{*}$ evaluates to 2B/δ in the interior regime and to G/δ in the boundary regime. Because in general $Q_{\max}<1$ in well-mixed populations, individuals continue to filter at the individually optimal rate $x^{*}$ even after the ceiling is reached, so the cultural pool remains pinned at $Q_{\max}$, and the payoff difference between social and individual learning becomes constant:

[34] $$\begin{array}{c} \Delta W_{\mathrm{sat}}=\delta \left(Q_{\max}-s+\rho \hat{\vartheta }x^{*}\right)-\left(L+c{\left(x^{*}\right)}^{2}\right). \end{array}$$

Here $c{\left(x^{*}\right)}^{2}$ takes the value B in the interior regime and c in the boundary regime. Whenever bias is worth expressing at all—that is, whenever B>L in the interior regime, or G>L+c in the boundary regime—one has $\Delta W_{\mathrm{sat}}>0$, so selection continues to favor social learning even after the information ceiling is hit (a short verification is given in *SI Appendix*). Unlike situations where efficiency costs stabilize traits at intermediate frequencies, here the absence of a negative feedback loop drives the population to fixation ($q^{*}\to 1$).

Achieving an interior rest point for q requires the presence of frequency-dependent costs for social learning. Consider the case where social learning costs are not constant but instead are proportional to social-learning frequency:

[35] $$\begin{array}{c} \ell =\ell_{0}+\gamma \;q, \end{array}$$

where $\ell_{0}$ is the baseline constant cost of social learning and γ is a proportionality constant capturing the effects of crowding or efficiency loss as social learning becomes more common. Several mechanisms can generate such crowding: depletion of a resource governed by the learned behavior, the opportunity cost of time spent observing rather than verifying through individual trial and error, and the devaluation of social information when proliferating copiers crowd out the individual learners who track environmental change (21). Our treatment captures all three in reduced form through γ, without committing to a particular mechanism.

This cost structure inflates the threshold $q^{†}$, making social learning harder to invade. The expression below is a first-order approximation valid when the threshold is small (L−B small); the exact quadratic root is derived in *SI Appendix*:

[36] $$\begin{array}{c} q_{\gamma >0}^{†}\approx \left\{\begin{array}{cc} \frac{L-B}{L+B-\gamma }, & L>B\\ 0, & L\leq B. \end{array}\right. \end{array}$$

This negative feedback also induces an interior rest point:

[37] $$\begin{array}{c} q^{*}={\text{clip}}_{\left[0,1\right]}\left(\frac{1-\delta \left(1-M^{*}\left(q\right)\right)-\left(\ell_{0}+c{\left(x^{*}\right)}^{2}+V_{\mathrm{IL}}\right)}{\gamma }\right). \end{array}$$

Assuming the saturated cultural pool affords perfect reliability ($M^{*}\approx 1$, at saturation under high absolute bias values), the mismatch penalty vanishes, yielding:

[38] $$\begin{array}{c} q_{\mathrm{sat}}^{*}={\text{clip}}_{\left[0,1\right]}\left(\frac{1-(\ell_{0}+c^{*}+V_{\mathrm{IL}})}{\gamma }\right), \end{array}$$

where

[39] $$\begin{array}{c} c^{*}=c\cdot {\left(x^{*}\right)}^{2}=\;\min\left(B,c\right) \end{array}$$

is the cost of optimal bias. The min is exactly the interior/boundary distinction: It returns $c^{*}=B$ when the optimum is interior (B≤c) and $c^{*}=c$ when the bias saturates at the boundary (B>c).

The term in the numerator of Eq. **37** is the net profit of social learning: its excess payoff over individual learning once costs of social exploration, mismatch penalty, and optimal bias are subtracted. Whenever this net profit exceeds the crowding multiplier γ social learning evolves to fixation (provided $q>q^{†}$). When it does not, an interior solution for $q^{*}$ emerges. Intuitively, the stable frequency of social learners decreases with increasing social learning costs ($\ell_{0}$, γ) and increasing payoffs for individual learning ($V_{\mathrm{IL}}$). The mismatch penalty δ enters $q^{*}$ through two channels that pull in opposite directions, and it is worth separating them. The direct effect runs through the bias-induced gain: A larger δ raises B (and G), making filtering more valuable; as long as B<c, this raises $q^{*}$, as does any increase in |ρ| or $\hat{\vartheta }$. The indirect effect runs through the mismatch term in $V_{\mathrm{SL}}$: A larger δ also makes each failed copy more costly, which lowers the payoff to social learning and hence $q^{*}$. The two effects coexist; for the equilibrium frequency the indirect effect dominates, so $q^{*}$ decreases on net with δ. An analogous relationship was found in our previous simulation model, where the distance between ecological niches θ, conceptually equivalent to this model’s δ, was found to decrease equilibrium social-learning frequency (18).

It is also worth mentioning that when $q^{*}<1$, $q_{\mathrm{sat}}$ becomes the Pareto optimum for social-learning frequency, yielding the highest mean fitness that can be achieved by the population. However, since $q_{\mathrm{sat}}<q^{*}$, populations will fail to reach this efficient equilibrium, instead evolving to the point in which the mean fitness of social learners equals that of pure individual learners, a classic result usually known as Rogers’ paradox (21).

**Fig. 4.**
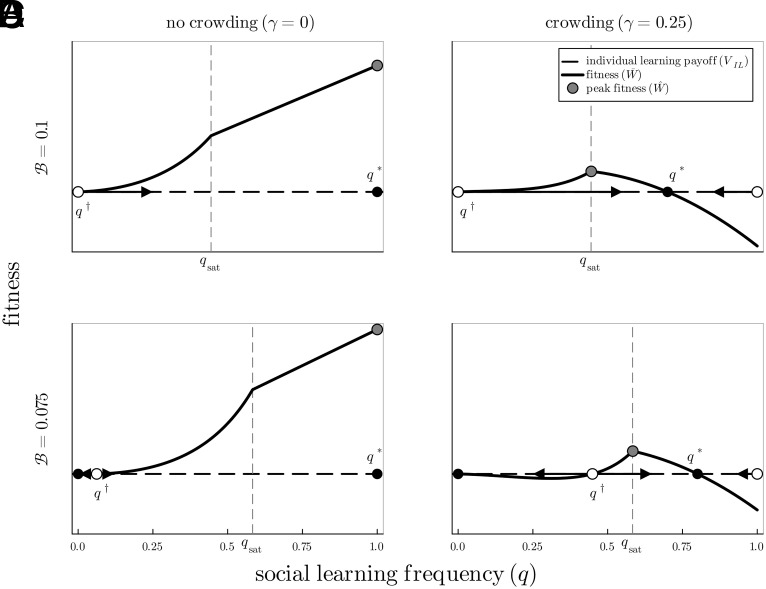
The effect of crowding costs and tag reliability on the evolution of social learning. Panels show the population fitness (W, black line) versus the payoff for pure individual learning ($V_{\mathrm{IL}}$, dashed black line) as a function of social learning frequency q. White dots represent unstable equilibria, black dots represent stable equilibria, and grey dots represent the peak of the mean fitness function. (*A*) (No crowding, γ=0): Social learning invades from rarity and sweeps to fixation (q→1), as there are no density-dependent costs to limit its spread. (*B*) (Crowding, γ>0): In the presence of crowding, invasion is possible if $q>q^{†}$, but the population stabilizes at an interior equilibrium $q^{*}$ where the informational benefits are matched by competitive costs. This stable point ($q^{*}$) occurs well past the Pareto optimum ($\hat{W}$). (*C*) As in A, there are no crowding costs, but social learning only invades given it starts past the threshold $q^{†}$. (*D*) As in B, crowding costs are present, but social learning only invades given it starts past the threshold $q^{†}$. Plotted for $l_{0}=0.015,\;c=0.15,\;V_{\mathrm{IL}}\approx 0.71$.

Fig. 4 displays phase plots for regimes with and without crowding costs for a high and low value of B. These trajectories demonstrate that, depending on whether there are crowding costs and how much information is available for bias to exploit at optimality, the population either undergoes runaway selection toward fixation (q→1) or stabilizes at an interior equilibrium ($q^{*}<1$) where the informational surplus of the cultural pool is offset by the competitive cost of accessing it.

### Risk Aversion Increases the Magnitude of Tag-Based Bias.

While the dynamics of finite populations generally mirror the infinite-population case, the increased importance of sampling variance creates a selection pressure against risky strategies. By maximizing the risk-adjusted fitness function, we derive an implicit condition for the optimal bias under evolutionarily induced risk aversion:

[40] $$\begin{array}{c} 2cx^{*}=\rho \;\hat{\vartheta }\;\delta \left[1+\frac{\delta }{N_{e}}\left(2M\left(x^{*}\right)-1\right)\right]. \end{array}$$

The term in the square brackets represents the risk adjustment factor that inflates the magnitude of optimal bias. To interpret this term, we examine the invasion condition where the cultural pool is determined by individual learners (q≈0, Q≈s). A mutant social learner utilizing optimal bias realizes an informational gain $\Delta_{M}=\rho \hat{\vartheta }x^{*}$, resulting in a success probability of $M^{*}\approx s+\Delta_{M}$. Substituting this into the expression for optimal bias yields:

[41] $$\begin{array}{c} x_{N_{e}}^{*}\approx x_{\infty }^{*}\left[1+\frac{\delta }{N_{e}}\left(2\left(s+\Delta_{M}\right)-1\right)\right]. \end{array}$$

Given the assumption that individual learning outperforms random guessing (s>1/2), and because the informational gain from optimal bias is nonnegative ($\Delta_{M}\geq 0$), the term $\left(2\left(s+\Delta_{M}\right)-1\right)$ is strictly positive. Consequently, the optimal bias in a finite population is strictly larger than in an infinite population, provided the unconstrained optimum remains interior; once the clip in Eq. **29** binds and $|x^{*}|=1$, risk aversion cannot increase the bias magnitude further. Risk-averse individuals increase their investment in the tag-based filter to minimize outcome variance. This inflation scales proportionally with the mismatch penalty δ, the accuracy of individual learning s, and the magnitude of the informational gain $\Delta_{M}$. The magnitude of parochialism or antisimilarity bias thus increases with both the mismatch penalty and the sampling variance of social transmission. Finite-population risk aversion operates through a second channel as well: It shifts the invasion threshold $q^{†}$ in a direction determined by the variance differential between social and individual learning. When social learning is the higher-variance strategy (the squared-penalty ratio $\delta^{2}/\beta^{2}$ exceeds the binomial-variance ratio $s\left(1-s\right)/[M^{*}\left(1-M^{*}\right)]$), $q^{†}$ rises and copying becomes harder to invade; when individual learning is the higher-variance strategy, $q^{†}$ falls. The bias-magnitude response of Eq. **41** and this threshold response are two distinct channels through which the same machinery operates; the full derivation is in *SI Appendix*.

Three implications follow. First, parochialism should be stronger in small, isolated communities than in large, well-mixed ones, since copying mistakes are less forgiving when sampling variance is high. Second, the same mechanism amplifies antisimilarity bias: Where a structurally disadvantaged tag is locally dominant, risk aversion can intensify assimilation as readily as it intensifies parochialism. Third, because the effect scales with the mismatch penalty δ, small communities should show especially pronounced filtering—parochial or antiparochial—where suboptimal behaviors are very costly.

## Discussion

Our demographic theory demonstrates that social identity can function as an informational tool in heterogeneous environments. Coupling the evolution of social learning with tag-based filtering biases helps to explain the transition from individual discovery to cultural accumulation. The necessity of similarity bias depends on copying costs: If unbiased social learning is cheaper than individual learning, filtering provides an additive advantage; if demographic diversity renders unbiased copying costlier, a parochial filter is required for cultural transmission to be viable.

The analytical framework presented here builds on our previous agent-based simulations (18), which relied on a particular topological arrangement of trait-space. The present treatment clarifies that the evolutionary driver of parochialism is the underlying informational contrast between in-group and out-group reliability, robust to both the topological details of the learning task and the specific mechanics of the filtering process, and extends the logic to antisimilarity bias. Our work also complements classic results by Boyd and Richerson (22) showing that if similarity-biased social learning is taken as given, tags will evolve to correlate with adaptive behaviors.

Our model further predicts a deterministic runaway to social-learning fixation in the absence of negative feedback mechanisms, whereas our previous simulated populations typically stabilized at intermediate frequencies (18). The difference arises because filtering in finite populations creates an implicit search cost that acts as a brake on fixation; the crowding-cost extension here models such a constraint. The analytical results without crowding therefore provide an optimistic upper bound on parochialism, especially for minority groups facing a trade-off between signal quality and sample size.

Before turning to these applications, we delineate what the model does and does not formalize. The framework isolates a single mechanism—the informational value of a group tag for a learner sampling a demographically mixed pool—and holds fixed several features of real populations: Tags are fixed rather than strategically alterable, fitness derives from a single behavioral domain, and tag reliability ρ is exogenous, with no endogenous feedback by which widespread assimilation or antisimilarity bias would itself reshape the marker–behavior correlation. These abstractions are what make the comparative statics tractable; they also mean that the empirical cases we discuss—majority–minority dynamics, the sociology of immigration, the persistence of colonial hierarchy—are best read not as direct explanations but as hypotheses that the model’s comparative statics suggest and help organize. We flag the most important of these limitations as they arise.

It is useful to keep in view a case in which the model’s mechanism operates cleanly: learning across age cohorts. Cohort members are typically more likely to share the constraints of their particular life stage, so an age tag carries real information about the local appropriateness of a behavior, and bias toward it reflects that information rather than prestige or coercion. This case is a useful reminder that the underlying mechanism we outline is general and does not depend on the additional dynamics of status and power, despite its utility in helping to understand them.

### The Cultural Multiplier and Evolutionary Stability.

A recurrent theme in cultural evolution is the startup problem: How does social learning become useful enough to invade a population of individual learners? Our analysis of the invasion regime identifies a specific synergy between copying and filtering. Social learning generates a “cultural memory” whose depth grows with the prevalence of copying: It is short when copiers are rare and the marginal benefit of filtering is low, and long when copying is common, so that the value of the similarity-biased filter grows with it.

This nonlinearity leads to frequency-dependent selection. For intermediate values of social learning costs and marker reliability, there is a sizable regime in which (similarity-biased) social learning is stable when common but fails to invade when rare. This echoes prior results for models in which selection on social learning exhibits positive frequency dependence by conditionally or critically supplementing individual learning (23–25). There the feedback typically requires learners to evaluate the content or success of what they observe; similarity bias supplies a structurally similar feedback more cheaply, conditioning on an observable tag rather than on an assessment of each target’s competence. The same qualitative bistability is expected for any rule that refines information above the individual-learning baseline under stationarity; what is distinctive to similarity bias is the informational channel it relies on.

This frequency dependence makes the invasion threshold a basin boundary: populations beginning below it collapse to individual learning while those beginning above it evolve to high copying frequencies. Our model characterizes the threshold but not the crossing, which requires some source of initial social-learning frequency—perturbation, gradual change in copying costs, migration—outside the present analysis. Once the threshold is crossed, the self-reinforcing cultural multiplier sustains a level of cultural fidelity unrecoverable from a population reset to pure individual learning.

### Risk Aversion and Parochialism.

Our model predicts that as the variance in payoffs increases, selection favors stricter filtering biases to minimize the risk of acquiring maladaptive information. As derived, this prediction operates through two channels of the same machinery: an increase in the magnitude of the optimal filtering bias—how selectively a learner conditions on tags when choosing whom to copy—and a shift in the invasion threshold for social learning, whose direction depends on the variance differential between social and individual learning. A separate exploration–exploitation logic governs the choice of whether to copy at all, and there the effect of risk can run the other way (26–28). Our model does not speak to that trade-off: The risk being captured is variance in the transmission of a fixed optimal behavior, not uncertainty about the outcome of that behavior. Within that narrower scope, our model indicates that, conditional on the availability of relevant social-learning strategies, riskier environments select for more stringent filtering of targets. The prediction aligns with claims from social and evolutionary psychology linking threat perception to in-group cohesion and out-group derogation (29). Research linked to Terror Management Theory, which posits that the salience of mortality drives individuals to cling more tightly to their cultural worldviews and hence to exhibit more in-group favoritism, is perhaps the most explicit example of this idea (e.g., ref. 30), though the findings supporting that theory have routinely failed to replicate (31–33). It is entirely possible that “mortality salience” (reminding people of potentially lethal threats) is simply too weak and proximate a stimulus, and too unconnected with the acquisition of useful information, to reflect differences in culturally evolved tendencies for parochial social learning. This inconsistency highlights the need for empirical research driven by formal theory to clarify predictions and delineate scope (34).

A broader empirical literature documents that perceived threat of several kinds tightens in-group preference. The “behavioral immune system” hypothesis holds that xenophobic attitudes function as a perceived prophylactic against novel pathogens carried by out-groups (35), and economic scarcity has been shown to narrow the perceived in-group (36). These findings are suggestive, but the connection to our model is by analogy rather than derivation. The risk our model formalizes is risk in the economic sense—variance in the outcome of a learning event—not predation, mortality, or environmental lethality. Economic variance is two-sided—high variance carries both downside risk and rare jackpots—and only the downside drives the risk-aversion result. Pathogen and mortality threat have no comparable upside. The shared logic therefore holds specifically for the loss side: When the cost of a bad outcome looms large, the adaptive response is to filter more stringently.

### Advantaged Majorities and the Privilege of Unbiased Learning.

When a group is both demographically dominant and possesses traits that are adaptive or status-conferring (Fig. 2, *Upper Right*), the model predicts that the evolutionary incentive for strong similarity bias declines, because the demographic leverage available to a filter is small: With few out-group targets in the pool, there is little for a filter to exclude. A majority learner can copy near-indiscriminately and rarely err. In this sense the regime characterizes the “unmarked” quality of dominant culture, in which the majority is exempted from the cost of maintaining a strategic filter.

A majority learner’s risk of maladaptive copying is the product of two quantities: the low probability of sampling an out-group target and the penalty incurred when such copying fails. Our negligible-risk conclusion therefore holds only when that penalty does not itself scale up with dominance. Where it does—as when a privileged majority faces a steep material or social penalty for copying a stigmatized minority—a small probability multiplied by a large cost need not be negligible, and the incentive to filter can survive even for subpopulations in the extreme majority.

In educational sociology, Carter (37) documents an analogous asymmetry: Minority students often develop the capacity to toggle between peer and school codes, while dominant-group students rarely develop comparable code-switching skill. This is a multidomain phenomenon, outside the scope of our single-domain framework, and we offer it as illustration rather than direct prediction. The “unmarked” character of dominant culture noted above also aligns with the sociological theory of unmarked categories (38, 39): Widely shared cultural traits cease to be signals of group membership and become the background baseline of the social world. A complementary approach to modeling multiple identity signals appears in ref. 40.

### Identitarian Defense and the Preservation of Ethnic Niches.

When a minority group possesses traits that are adaptive within a specific niche but is demographically dispersed (Fig. 2, *Lower Right*), the optimal strategy is strong similarity bias. Without bias, the minority learner faces a high risk of signal dilution: Random copying would rapidly replace specialized in-group traits with generalist or maladaptive out-group traits. Indeed, this is the parameter regime most strongly associated with similarity-biased social learning, and therefore predicts that such a parochial bias will be most prevalent in minority communities or subcultures in which strong identification is needed to maintain adaptive cultural cohesion.

This dynamic connects to sociological theories of selective acculturation (41), in which immigrant groups maintain socioeconomic advantage through strict internal norms rather than wholesale adoption of host-population behavior. The connection holds, in the model’s terms, only when in-group membership is genuinely predictive of the locally appropriate behavior for the learner, because the optimal action itself differs by group, not merely because group identity gates access to a resource. Consider a recent migrant deciding what car to buy or which financial commitments to take on: The right answer depends on visa status, credit access, and the regulatory treatment of noncitizens—conditions shared with other migrants in similar legal positions and not with the general population. Here in-group tags genuinely predict the locally adaptive behavior, and strong similarity bias is the model’s prediction.

The dynamic extends to any “local minority” possessing a perceived advantage, whether colonial administrators clinging to home-country norms more rigidly than in the metropole (42) or modern expatriate communities insulating their networks from the host society (43, 44). While such patterns may partly reflect the ease of forming ties with similar others, they also ensure that any status differential driving local privilege is not diluted by assimilation.

### Active Assimilation and the Lasting Consequences of Colonialism.

When a group carries a trait negatively correlated with payoffs—due to structural stigma, technological obsolescence, or hegemonic devaluation—but is locally ubiquitous (Fig. 2, *Upper Left*), selection shifts toward active rejection of information from the in-group and a preference for information from the (majority) out-group. While it is commonly assumed that segregation promotes cultural retention, our model illustrates conditions in which segregation can actually accelerate the strategic abandonment of identity.

In this context, the learner faces an inverted signal-to-noise problem. The “noise” (the maladaptive in-group trait) is not rare; it is the overwhelming default environment. However, the majority-group learner possesses a particular informational advantage: The ubiquity of the “error” provides a high-fidelity template for calibration. By adopting a strategy of strong antisimilarity bias and preferentially learning from the out-group, an individual can locate adaptive out-group traits with high efficiency.

In our model, tag reliability encodes the correlation between tag and payoff, with payoff defined by whatever the prevailing incentive structure rewards. When proximity to a dominant group’s behavior confers access to labor markets, legal standing, capital, or marriage prospects, that behavior is the higher-payoff option for the learner. If that dominant group is reliably marked and in the minority, its markers can confer a sort of prestige that can emerge through a bias for learning from out-group individuals for those in the subordinate majority. Our model does not explain how such an incentive structure arises: It treats tag reliability as exogenous. In colonial and postcolonial settings, the relationship between marker and payoff emerges endogenously through processes of domination, with cultural transmission contributing directly to the persistence of outdated perceptions of incentive structures long after the conditions that engendered them have changed (45–47). Without such external forces, antisimilarity bias would be self-undermining—widespread out-group copying would erode the marker–behavior correlation. The correlation will persist only when processes like continued group domination reinforce the skew faster than assimilation can erase it.

This relationship describes several documented social patterns. Second-generation immigrants in marginalized communities sometimes engage in hyperassimilation, to the point of holding deprecating views of the parental culture (48–50). The model’s logic also bears on the persistence of colonial hierarchies in postindependence nation-states (51, 52): Indigenous or Afro-descendant populations, despite often constituting the demographic majority, may function as “minoritized” subjects whose traits are devalued in public discourse, an inversion compounded by the colonial dismantling of precolonial institutions that had conferred local benefits. Where the postcolonial incentive structure continues to reward proximity to the formerly colonial group, the model predicts antisimilarity bias even among a demographic majority. The phenomenon of *colorism* and skin whitening (53, 54)—in which individuals across the Global South alter their skin tone toward a Eurocentric ideal—is an acute expression, as is the pattern Vigoya (55) documents among the Black middle class in Colombia, where upward class mobility is often coupled with the shedding of racial markers.

### Passive Assimilation and the Immigrant Paradox.

Finally, we consider the regime where a minority group is demographically dispersed and faces a structural penalty or opportunity cost for their local traits (Fig. 2, *Lower Left*). Our model predicts that in this case, minority group members will assimilate not by actively rejecting their identity, but by passively ceasing to defend it against the statistical weight of the majority. Because their environment is already information-rich with out-group models, the optimal learning strategy is simply weak or neutral bias. For example, minority children in mixed institutions often lose their heritage language and cultural competence with remarkable speed (56). The loss need not be driven by active self-deprecation, but by the ubiquity of the dominant culture in the peer environment, which renders the maintenance of the mother tongue prohibitively costly without a strict protective filter (57).

Both active and passive assimilation may also bear on the so-called “immigrant paradox,” the pattern in which recent immigrants to the United States often show better health and social profiles than their U.S.-born descendants (58–61). A single-payoff model cannot represent the paradox directly: A heritage trait could be disfavored on the dimension tag reliability tracks—status, credentialing, economic integration—while remaining protective on a dimension the model does not represent, such as health. We offer this as a connection the model suggests rather than derives; treating it properly would require a multidomain extension.

### Limitations and Future Directions.

Our model abstracts away several complexities to achieve analytical tractability. First, we assumed that fitness arises from a single behavioral domain. In reality, learners acquire many behaviors across many domains, and the tag-payoff correlation need not have the same sign or magnitude in each. A learner may belong to a group that is a reliable source for one class of behaviors but a misleading source for another: A teenager might gain by copying an age-mate’s taste in music while losing by copying that same age-mate’s inexperienced driving.

Our single-domain treatment is shared by the vast majority of social-learning models, but it is still unlikely that humans adopt a uniquely optimal strategy for every domain. Similarity-biased social learning may instead be one input to more general learning heuristics that optimize across the expected range of domains rather than for any single instance (62, 63). Extending the model to multiple domains with potentially distinct values of ρ—and allowing learners to (mis)generalize a tag’s reliability across them—is a natural next step.

Second, we treated tags as fixed cues of identity. In reality, identities evolve, and tags are themselves cultural traits that can be copied, disguised, or strategically altered. If migrants can adopt local tags to “pass” as natives, for example, the correlation between tag and adaptive behavior would degrade. This sets the stage for a coevolutionary arms race between signalers (who may or may not want to be correctly identified) and learners (who want reliable tags), potentially destabilizing the conditional strategy equilibrium (64).

Finally, we focused on a static environment where the “correct” behaviors are fixed. This assumption is what allows both the runaway social-learning fixation and the cultural-multiplier-driven bistability to operate. In a temporally varying environment, the “cultural memory” that aids accumulation becomes a liability, as it preserves outdated information (47, 65). The interplay between the runaway dynamics derived here and environmental volatility is thus a viable extension. For example, future work could examine whether environmental change imposes a check on the runaway process—forcing the population back to an intermediate frequency of social learning—and its effects on the incentives for similarity bias. Another interesting question is whether similarity bias impedes populations from abandoning traditionalism quickly enough to avoid extinction. Relatedly, our dynamics assume that strategy frequencies change in proportion to payoff differences and that the learning decision is made once per generation. Both are near-universal simplifications in formal models of cultural evolution, but neither is innocuous: Payoff-proportional dynamics are only one of many possible processes for cultural selection (66), and treating learning as a once-per-generation event understates the contributions of development and exploration.

Nevertheless, we have provided a baseline for further exploration of similarity-biased social learning. Considering that science is a collective enterprise under a broad tent, we hope the reader considers us worth learning from.

## Materials and Methods

We developed a model in which individuals choose between individual learning and social learning biased by an identity tag toward either same-tag or different-tag demonstrators. Derivations of model results are given in *SI Appendix*. Numerical results throughout the paper were obtained using the Julia language (67); a Pluto notebook (68) that reproduces the plots is available at https://github.com/datadreamscorp/DemographicSimilarityBias (69). An earlier version can be found in ref. 69.

## Supplementary Material

Appendix 01 (PDF)

## Data Availability

Previously published data were used for this work [Numerical results throughout the paper were obtained using scripts written in the Julia programming language (67); a Pluto notebook (68) that reproduces the plots is available at https://github.com/datadreamscorp/DemographicSimilarityBias (69)].

## Footnotes

^*^The variance-penalized criterion of ref. 20 derives from the compounding of fitness across generations: A strategy with higher within-generation variance accumulates a lower geometric mean growth rate over evolutionary time. The criterion therefore presumes that selection operates through multigenerational compounding. If strategies instead spread through a within-generation process—repeated payoff-biased choice among learning rules within a single lifetime—the qualitative direction of the finite-population effect (stronger filtering when social learning is the lower-variance strategy) should be robust, but the precise $\sigma^{2}/N_{e}$ form is specific to the compounding interpretation.

^†^The extreme case in which the ceiling already lies below the individual-learning baseline, $Q_{\max}\left(\rho ,f\right)<s$—which arises when the available demographic leverage and tag reliability are jointly large enough that $\left|\rho \right|\hat{\vartheta }>1-s$—is handled in *SI Appendix*. There the feasibility projection pins the pool at $Q_{\max}$ for all q, the selection gradient on q becomes frequency-independent, and the results of this section carry over under the substitution $L\to L+\delta (|\rho |\hat{\vartheta }-\left(1-s\right))$, with no bistability.
