# Supplementary material for: A demographic theory of similarity-biased social learning
Source: Proc Natl Acad Sci U S A. 2026 Jul 30;123(31):e2606741123. doi: 10.1073/pnas.2606741123 (PMC13438531; doi:10.1073/pnas.2606741123)
Supplement: Supplementary file 1 [file pnas.2606741123.sapp.pdf]

Supplementary Information

A demographic theory of similarity-biased learning

Alejandro Pérez Velilla[1,2,*], Paul E. Smaldino[1,3,4]

[1] University of California, Merced
[2] Max Planck Institute for Evolutionary Anthropology
[3] Santa Fe Institute, Santa Fe, NM
[4] Complexity Science Hub, Vienna, Austria
[*] Corresponding author: Alejandro Pérez Velilla,
aperezvelilla@ucmerced.edu

A. Derivation of conditional accuracies

Let C denote the event that a randomly sampled demonstrator exhibits the optimal behavior, such that the prior probability of this event is the aggregate cultural accuracy, $P(C) = Q_t$. Let S denote the event that a demonstrator shares the learner's tag (in-group), with prior probability $P(S) = f$, and let O denote the complementary event of an out-group demonstrator, with $P(O) = 1 - f$.

The conditional accuracies of the two demographic groups, $p_{s,t}$ and $p_{o,t}$, are the posterior probabilities of observing correct behavior given the target's identity:

$$p_{s,t} = P(C|S) \quad [42]$$

$$p_{o,t} = P(C|O) \quad [43]$$

By the Law of Total Probability, the marginal probability of sampling the correct behavior from the population is the sum of the conditional probabilities weighted by their group frequencies:

$$P(C) = P(C|S)P(S) + P(C|O)P(O)\,. \quad [44]$$

Substituting the model parameters into this expansion yields:

$$Q_t = p_{s,t} f + p_{o,t}(1 - f)\,. \quad [45]$$

Tag reliability ρ is defined as the difference between these two posterior probabilities:

$$\rho = P(C|S) - P(C|O) = p_{s,t} - p_{o,t}\,. \quad [46]$$

To solve for the conditional accuracy of same-tag demonstrators, $p_{s,t}$, we rearrange the reliability definition to $p_{o,t} = p_{s,t} - \rho$ and substitute it into the total probability expression:

$$\begin{aligned} Q_t &= p_{s,t} f + (p_{s,t} - \rho)(1 - f) \\ Q_t &= p_{s,t} f + p_{s,t} - p_{s,t} f - \rho(1 - f) \\ Q_t &= p_{s,t} - (1 - f)\rho \end{aligned} \quad [47]$$

Rearranging for $p_{s,t}$ yields the substitution for an in-group target:

$$p_{s,t} = Q_t + (1 - f)\rho\,. \quad [48]$$

Applying the symmetric substitution ($p_{s,t} = p_{o,t} + \rho$) into the total probability equation provides the substitution for an out-group target:

$$p_{o,t} = Q_t - f\rho\,. \quad [49]$$

B. Tag reliability as a regression coefficient

We asserted that ρ is equivalent to the regression coefficient of behavioral correctness on tag identity. Let Y be a binary variable for behavioral correctness ($1 =$ correct, $0 =$ incorrect) and X be a binary variable for tag identity relative to the learner ($1 =$ same tag, $0 =$ other tag).

The ordinary least squares regression coefficient β_{YX} is given by:

$$\beta_{YX} = \frac{\mathrm{Cov}(X, Y)}{\mathrm{Var}(X)} = \frac{\mathbb{E}[XY] - \mathbb{E}[X]\mathbb{E}[Y]}{\mathbb{E}[X^2] - (\mathbb{E}[X])^2} \quad [50]$$

Given the frequencies f (same tag) and $1 - f$ (other tag):

$$\mathbb{E}[X] = 1 \cdot f + 0 \cdot (1 - f) = f \quad [51]$$

$$\mathrm{Var}(X) = f(1 - f) \quad [52]$$

The conditional expectations of Y are $\mathbb{E}[Y|X = 1] = p_{s,t}$ and $\mathbb{E}[Y|X = 0] = p_{o,t}$. The term $\mathbb{E}[XY]$ represents the probability that a target is both same-tag and correct:

$$\mathbb{E}[XY] = f \cdot p_{s,t} \quad [53]$$

The term $\mathbb{E}[Y]$ is simply the population accuracy Q_t. Substituting these into the covariance:

$$\begin{aligned} \mathrm{Cov}(X, Y) &= f p_{s,t} - f Q_t \\ &= f p_{s,t} - f[f p_{s,t} + (1 - f)p_{o,t}] \\ &= f p_{s,t}(1 - f) - f(1 - f)p_{o,t} \\ &= f(1 - f)(p_{s,t} - p_{o,t}) \end{aligned} \quad [54]$$

Thus, the regression coefficient is:

$$\beta_{YX} = \frac{f(1 - f)(p_{s,t} - p_{o,t})}{f(1 - f)} = p_{s,t} - p_{o,t} = \rho \quad [55]$$

C. Derivation of optimal bias (x^*)

To find the optimal bias, we maximize the invasion fitness of a mutant strategist with respect to bias x'. In an infinite population, the relevant component of the fitness function W_{mut} is the expected payoff to social learning minus the cost of bias:

$$V_{\mathrm{SL}}(x') = 1 - \delta[1 - M(Q, x')] - c(x')^2 - \ell\,. \quad [56]$$

Recall that $M(Q, x') = Q + \rho\vartheta(x')x'$. Because the demographic leverage $\vartheta(x')$ takes different values for $x' > 0$ and $x' < 0$, the payoff V_{SL} is piecewise quadratic with a kink at $x' = 0$, and the derivative there is two-sided. On each open branch the derivative is

$$\frac{\partial V_{\mathrm{SL}}}{\partial x'} = \delta\rho\vartheta(x') - 2cx'\,, \quad [57]$$

so the one-sided derivatives at the kink are

$$\left.\frac{\partial V_{\mathrm{SL}}}{\partial x'}\right|_{x' \to 0^+} = \delta\rho(1 - f)\,, \qquad \left.\frac{\partial V_{\mathrm{SL}}}{\partial x'}\right|_{x' \to 0^-} = \delta\rho f\,. \quad [58]$$

Both one-sided derivatives carry the sign of ρ. The point $x' = 0$ is therefore not an ordinary interior stationary point: when $\rho > 0$ both are positive, so V_{SL} is increasing through the kink and the optimum lies strictly in $x' > 0$; when $\rho < 0$ both are negative, so the optimum lies strictly in $x' < 0$; and only when $\rho = 0$ do both vanish, making $x' = 0$ optimal. Within the relevant branch V_{SL} is smooth and strictly concave (second derivative $-2c < 0$), so the interior optimum is found by setting the branch derivative to zero:

$$x^* = \frac{\delta\rho\vartheta(x^*)}{2c}\,. \quad [59]$$

Since the sign of x^* matches the sign of ρ (for $\delta, c > 0$), the leverage evaluated at the optimum is $\hat{\vartheta} \equiv \vartheta(x^*)$, equal to $1 - f$ when $\rho > 0$ and f when $\rho < 0$. This yields the optimal bias of main text Eq. (18), and the case-split in the bias formula is a direct consequence of the jump in ϑ at the kink rather than an arbitrary convention.

In finite populations, the optimization must incorporate the variance penalty. The risk-adjusted fitness of a mutant social learner is:

$$W_{\mathrm{SL}}(x') = V_{\mathrm{SL}}(x') - \frac{1}{N_e}\sigma^2_{\mathrm{SL}}(x')\,. \quad [60]$$

Substituting the variance of a social learner, $\sigma^2_{\mathrm{SL}} = \delta^2 M(Q, x')[1 - M(Q, x')]$:

$$W_{\mathrm{SL}}(x') = 1 - \delta[1 - M(Q, x')] - c(x')^2 - \ell - \frac{\delta^2}{N_e}M(Q, x')[1 - M(Q, x')]\,. \quad [61]$$

Taking the partial derivative with respect to x' requires applying the chain rule to the variance term:

$$\begin{aligned} \frac{\partial W_{\mathrm{SL}}}{\partial x'} &= \delta\frac{\partial M}{\partial x'} - 2cx' - \frac{\delta^2}{N_e}\left(\frac{\partial M}{\partial x'} - 2M(Q, x')\frac{\partial M}{\partial x'}\right) \\ &= \delta\rho\vartheta(x') - 2cx' - \frac{\delta^2}{N_e}[1 - 2M(Q, x')]\rho\vartheta(x')\,. \end{aligned} \quad [62]$$

Setting the gradient to zero, calling the finite population equilibrium $x^*_{N_e}$, and factoring out $\delta\rho\vartheta(x^*_{N_e})$:

DRAFT

$$2cx^*_{N_e} = \delta\rho\vartheta(x^*_{N_e})\left[1 - \frac{\delta}{N_e}[1 - 2M(Q, x^*)]\right] . \qquad [63]$$

Rearranging the inner terms and substituting $\hat{\vartheta}$ yields the implicit condition for the evolutionarily-induced risk-averse bias of the main text:

$$2cx^*_{N_e} = \delta\rho\hat{\vartheta}\left[1 + \frac{\delta}{N_e}(2M(x^*) - 1)\right] . \qquad [64]$$

In the invasion limit $q \to 0$, the cultural pool is determined by individual learners ($Q \to s$). A social learner utilizing optimal bias realizes an informational gain $\Delta_M = \rho\hat{\vartheta}x^*$, such that $M \to s + \Delta_M$. Solving for $x^*_{N_e}$ yields the explicit approximation of main text Eq. (41):

$$x^*_{N_e} \approx x^*_\infty\left[1 + \frac{\delta}{N_e}(2(s + \Delta_M) - 1)\right] \qquad [65]$$

where x^*_∞ is the optimal bias when $N_e \to \infty$.

D. Derivation of the net payoff gain ($\mathcal{B}$)

$\mathcal{B}$ is defined as the fitness difference between a social learner using optimal bias x^* and an unbiased social learner ($x = 0$), neglecting the fixed cost ℓ which applies to both.

$$\begin{aligned}\mathcal{B} &= V_{\mathrm{SL}}(x^*) - V_{\mathrm{SL}}(0)\\ &= [-\delta(1 - M(x^*)) - c(x^*)^2] - [-\delta(1 - M(0)) - 0]\\ &= \delta[M(x^*) - M(0)] - c(x^*)^2 . \end{aligned} \qquad [66]$$

Substituting $M(x^*) - M(0) = \rho\hat{\vartheta}x^*$:

$$\mathcal{B} = \delta\rho\hat{\vartheta}x^* - c(x^*)^2 . \qquad [67]$$

Substituting $x^* = \frac{\delta\rho\hat{\vartheta}}{2c}$:

$$\begin{aligned}\mathcal{B} &= \delta\rho\hat{\vartheta}\left(\frac{\delta\rho\hat{\vartheta}}{2c}\right) - c\left(\frac{\delta\rho\hat{\vartheta}}{2c}\right)^2\\ &= \frac{(\delta\rho\hat{\vartheta})^2}{2c} - \frac{(\delta\rho\hat{\vartheta})^2}{4c} = \frac{(\delta\rho\hat{\vartheta})^2}{4c} . \end{aligned} \qquad [68]$$

The closed form $\mathcal{B} = (\delta\rho\hat{\vartheta})^2/(4c)$ derived here is the *interior* expression: it follows from substituting the interior optimum $x^* = \delta\rho\hat{\vartheta}/(2c)$, and is valid only when that optimum satisfies $|x^*| \leq 1$. When the unconstrained optimum exceeds 1 in magnitude, the clip in main text Eq. (29) binds, x^* saturates at $|x^*| = 1$, and the general expression $\mathcal{B} = \delta\rho\hat{\vartheta}x^* - c(x^*)^2$ must be evaluated at the boundary instead. Setting $|x^*| = 1$ gives the boundary-regime payoff gain $\delta|\rho|\hat{\vartheta} - c$, the gross informational gain net of the maximal filtering cost. The main text writes the gross informational gain as $\mathcal{G} \equiv \delta|\rho|\hat{\vartheta}$ and carries both regimes through the subsequent results via the substitution $2\mathcal{B} \to \mathcal{G}$, $\mathcal{L} + \mathcal{B} \to \mathcal{L} + c$. All derivations in this supplement are stated for the interior regime unless noted; the boundary-regime counterpart of each follows by that substitution.

E. Derivation of evolutionary equilibria for social learning frequency

Here we derive the stationary states of the evolutionary dynamics governing the frequency of social learning (q). We partition the state space into two regimes: the interior regime, where cultural accuracy Q is strictly within the feasibility bounds imposed by demographic mixing, and the saturated regime, where accuracy is pinned at the theoretical maximum $Q_{\max}$. Throughout this section we carry over the main text assumption of $Q_{\max} \geq s$. In the following appendix section we show what happens once this assumption is relaxed, allowing for $Q_{\max} < s$.

Invasion threshold ($q^\dagger$). When the cultural pool is below the feasibility ceiling ($Q(q, x) < Q_{\max}$), we substitute the optimal bias x^* into the selection gradient for social learning to determine the equilibrium copying frequency. From the main text definitions of the baseline disadvantage of unbiased learning ($\mathcal{L}$) and the net payoff gain from bias ($\mathcal{B}$), the selection gradient simplifies to main text Eq. (23):

$$w_q(q) = \frac{2\mathcal{B}}{1 - q} - (\mathcal{L} + \mathcal{B}) . \qquad [69]$$

Stationarity requires $w_q(q) = 0$. Solving for q yields the invasion threshold $q^\dagger$ (main text Eq. (32)):

$$\begin{aligned}\frac{2\mathcal{B}}{1 - q^\dagger} &= \mathcal{L} + \mathcal{B}\\ 1 - q^\dagger &= \frac{2\mathcal{B}}{\mathcal{L} + \mathcal{B}}\\ q^\dagger &= \frac{\mathcal{L} - \mathcal{B}}{\mathcal{L} + \mathcal{B}} . \end{aligned} \qquad [70]$$

If $\mathcal{L} \leq \mathcal{B}$, the benefit of optimal bias exceeds the baseline disadvantage, and social learning invades unconditionally from zero ($q^\dagger = 0$). If $\mathcal{L} > \mathcal{B}$, the threshold is strictly positive, creating bistability: populations with $q < q^\dagger$ collapse to individual learning, while those with $q > q^\dagger$ evolve toward higher copying rates.

Saturation threshold (q_{sat}). The system enters the saturated regime when the frequency of social learning is high enough to drive the cultural pool to its feasibility limit, $Q_{\max} = \min\{1 + f\rho, 1 - (1 - f)\rho\}$. We define the saturation threshold q_{sat} as the minimum frequency of social learning required to reach this accuracy ceiling given the fixed optimal bias x^*. Using the interior expression for accuracy:

$$s + \frac{q_{\mathrm{sat}}}{1 - q_{\mathrm{sat}}}\rho\hat{\vartheta}x^* = Q_{\max} . \qquad [71]$$

Solving for q_{sat} yields main text Eq. (33):

$$\begin{aligned}\frac{q_{\mathrm{sat}}}{1 - q_{\mathrm{sat}}} &= \frac{Q_{\max} - s}{\rho\hat{\vartheta}x^*}\\ q_{\mathrm{sat}} &= \frac{\frac{Q_{\max} - s}{\rho\hat{\vartheta}x^*}}{1 + \frac{Q_{\max} - s}{\rho\hat{\vartheta}x^*}}\\ q_{\mathrm{sat}} &= \frac{Q_{\max} - s}{Q_{\max} - s + \rho\hat{\vartheta}x^*} . \end{aligned} \qquad [72]$$

This expression is regime-neutral in x^*: the product $\rho\hat{\vartheta}x^*$ evaluates to $2\mathcal{B}/\delta$ in the interior regime and to $\mathcal{G}/\delta$ in the boundary regime, so q_{sat} requires no separate derivation for the two cases.

Runaway dynamics. For all $q > q_{\mathrm{sat}}$, the cultural pool is pinned at $Q_{\max}$. Because individuals maximize private fitness, they do not relax their filtering bias x^* in this regime. Consequently, the payoff difference between social and individual learning becomes constant and frequency-independent:

$$\Delta W_{\mathrm{sat}} = \delta(Q_{\max} - s + \rho\hat{\vartheta}x^*) - (\mathcal{L} + c(x^*)^2) . \qquad [73]$$

If $\Delta W_{\mathrm{sat}} > 0$, the selection gradient is positive for all $q > q_{\mathrm{sat}}$. The absence of negative density dependence prevents the formation of an intermediate equilibrium, and selection drives the population to fixation ($q \to 1$), where the population becomes fully reliant on social learning.

We now verify that $\Delta W_{\mathrm{sat}} > 0$ holds precisely under the condition that makes bias worth expressing in the first place. The argument runs through the interior regime; the boundary regime follows by the substitution given below. Two facts constrain the terms of Eq. (73). First, the bias-induced informational gain is non-negative, $\rho\hat{\vartheta}x^* \geq 0$, because x^* carries the sign of ρ; in the interior regime it equals exactly $\rho\hat{\vartheta}x^* = 2\mathcal{B}/\delta$, since $x^* = \delta\rho\hat{\vartheta}/(2c)$ gives $\delta\rho\hat{\vartheta}x^* = (\delta\rho\hat{\vartheta})^2/(2c) = 2\mathcal{B}$. Second, this section assumes that $Q_{\max} \geq s$ (the case where $Q_{\max} < s$ is treated in F), so the gap $Q_{\max} - s$ is a non-negative bonus. Substituting the interior identities $\delta\rho\hat{\vartheta}x^* = 2\mathcal{B}$ and $c(x^*)^2 = \mathcal{B}$ into Eq. (73):

$$\Delta W_{\mathrm{sat}} = \delta(Q_{\max} - s) + 2\mathcal{B} - \mathcal{L} - \mathcal{B} = \delta(Q_{\max} - s) + (\mathcal{B} - \mathcal{L}) . \qquad [74]$$

Since $\delta(Q_{\max} - s) \geq 0$, it follows that $\Delta W_{\mathrm{sat}} \geq \mathcal{B} - \mathcal{L}$, and therefore

$$\mathcal{B} > \mathcal{L} \implies \Delta W_{\mathrm{sat}} > 0 . \qquad [75]$$

The condition $\mathcal{B} > \mathcal{L}$ is exactly the condition under which the invasion threshold vanishes ($q^\dagger = 0$) and biased social learning invades from rarity. Whenever bias is worth expressing, the saturated payoff gap is strictly positive, and the runaway to fixation follows. In the boundary regime the same argument applies under the substitutions $2\mathcal{B} \to \mathcal{G}$ and $\mathcal{L} + \mathcal{B} \to \mathcal{L} + c$, giving $\Delta W_{\mathrm{sat}} = \delta(Q_{\max} - s) + (\mathcal{G} - \mathcal{L} - c)$ and hence $\mathcal{G} > \mathcal{L} + c \Rightarrow \Delta W_{\mathrm{sat}} > 0$. Note that ΔW_{sat} can also be positive when the invasion threshold is itself positive ($\mathcal{B} \leq \mathcal{L}$), provided the feasibility bonus $\delta(Q_{\max} - s)$ is large enough; the implication established here is the one the main text relies on, namely that a population which can invade from rarity necessarily runs away to fixation once it saturates.

DRAFT

F. The constrained regime: $Q_{\max} < s$

The saturation analysis in the main text is stated for the regime where the feasibility ceiling $Q_{\max}(\rho, f)$ lies at or above the individual-learning baseline s, so that the cultural pool climbs from s up to $Q_{\max}$ as the frequency of social learning rises through $q_{\rm sat}$. This appendix treats the extreme case $Q_{\max}(\rho, f) < s$, in which the ceiling lies below the individual-learning baseline from the outset and saturation holds throughout. Using the identity $1 - Q_{\max}(\rho, f) = |\rho|\hat{\vartheta}$ (valid on either side of $\rho = 0$, with $\hat{\vartheta} = 1 - f$ for $\rho > 0$ and $\hat{\vartheta} = f$ for $\rho < 0$), the regime is characterized by

$$|\rho|\hat{\vartheta} > 1 - s. \tag{76}$$

We show that the dynamics in this regime are a specialization of the saturated dynamics of the main text: the cultural pool is pinned at $Q_{\max}$ for all q, the selection gradient on q is constant, and bistability does not arise. The invasion conditions in main text Eq. (32) carry over under a single substitution that adjusts for the fact that the unbiased pool sits at $Q_{\max}$ rather than at s.

Cultural pool. At $q = 0$, the unconstrained fixed point of the cultural recursion is $Q^*(0, x) = s$, which exceeds $Q_{\max}$ in this regime. The feasibility projection introduced in the main text pins the realized pool at $Q_{\max}$. For $q > 0$ with bias aligned with ρ, the unconstrained fixed point under biased copying is

$$Q^*(q, x) = s + \frac{q}{1-q}\,|\rho||x|\hat{\vartheta} \;\geq\; s \;>\; Q_{\max}, \tag{77}$$

so the projection clips the pool at $Q_{\max}$ for every $q \in [0, 1)$:

$$Q(q, x) = Q_{\max}(\rho, f) \qquad \text{for all } q \in [0, 1). \tag{78}$$

Social learner accuracy. With the pool pinned at $Q_{\max}$, the social learner's accuracy under bias aligned with ρ is

$$M(x) = Q_{\max} + |\rho|\hat{\vartheta}|x| = (1 - |\rho|\hat{\vartheta}) + |\rho|\hat{\vartheta}|x|. \tag{79}$$

At the boundary $|x^*| = 1$, $M = 1$: the targeted group is a perfect signal at saturation, consistent with the conditional probability of correctness reaching 1 for the favored group when the pool sits at the ceiling.

Optimal bias. Because $V_{\rm SL}$ retains the same functional form as in the main text—the difference is only in the constant value of Q entering M—the first-order condition $\partial V_{\rm SL}/\partial x' = 0$ yields the same expression

$$x^* = \frac{\delta\,\rho\,\hat{\vartheta}}{2c}, \tag{80}$$

with the interior/boundary distinction governed, as before, by whether the unconstrained value lies in $[-1, 1]$. The bias-induced gain $\mathcal{B} = (\delta\,\rho\,\hat{\vartheta})^2/(4c)$ and the boundary-regime gross gain $\mathcal{G} = \delta|\rho|\hat{\vartheta}$ retain their closed forms.

Selection gradient on social learning. Because Q is constant in q, the social learner's mean payoff $V_{\rm SL}(x^*)$ is also constant in q. The selection gradient on social learning,

$$w_q(q) = V_{\rm SL}(x^*) - V_{\rm IL}, \tag{81}$$

is therefore frequency-independent. There is no q-dependent term, no $q^\dagger$ as a basin boundary, and no $q_{\rm sat}$ as the frequency at which the pool first reaches the ceiling: the pool is at the ceiling from the start.

Invasion condition. The disadvantage of unbiased social learning relative to individual learning is now computed against a pool of accuracy $Q_{\max}$, not s. Letting $\mathcal{L}'$ denote this constrained-regime disadvantage:

$$\begin{aligned}
\mathcal{L}' &\equiv V_{\rm IL} - V_{\rm SL}(0) \\
&= \ell - k + \delta(1 - Q_{\max}) - \beta(1 - s) \\
&= \mathcal{L} + \delta\left(|\rho|\hat{\vartheta} - (1 - s)\right),
\end{aligned} \tag{82}$$

where the last line uses $1 - Q_{\max} = |\rho|\hat{\vartheta}$ and the definition $\mathcal{L} \equiv \ell - k - (\beta - \delta)(1 - s)$ from the main text. Because the constrained regime is defined by $|\rho|\hat{\vartheta} > 1 - s$ (Eq. (76)), we have $\mathcal{L}' > \mathcal{L}$: unbiased social learning is more disadvantaged than the standard formula suggests, reflecting that the unbiased copier samples a pool of accuracy $Q_{\max} < s$.

The invasion condition takes the same form as in the main text, with $\mathcal{L}$ replaced by $\mathcal{L}'$:

$$\text{interior:}\quad \mathcal{B} > \mathcal{L}', \tag{83}$$

$$\text{boundary:}\quad \mathcal{G} > \mathcal{L}' + c. \tag{84}$$

Whenever this condition is satisfied, the constant positive gradient drives social learning to fixation ($q \to 1$). When it is not, $q = 0$ is globally attracting and social learning never invades.

Comparison with the saturated $Q_{\max} \geq s$ regime in the main text. The constrained regime differs from the main-text saturated regime in three ways:

1. Saturation begins at $q = 0$ rather than at $q_{\rm sat} > 0$, so $q_{\rm sat}$ is degenerate and the dynamics are saturated throughout.
2. The unbiased baseline is $Q_{\max}$ rather than s, so the disadvantage of unbiased copying is $\mathcal{L}'$ rather than $\mathcal{L}$.
3. The selection gradient on q is constant, so the bistability and basin structure of the interior dynamics do not arise: invasion is unconditional in the parameter space defined by the inequalities above.

The interior/boundary distinction, the closed forms of $\mathcal{B}$ and $\mathcal{G}$, the substitution rule $2\mathcal{B} \to \mathcal{G}$, $\mathcal{L} + \mathcal{B} \to \mathcal{L} + c$, and the runaway-to-fixation result all carry over. Adding density-dependent crowding costs to the constrained regime yields an interior equilibrium q^* following the same derivation as in main text Eq. (37), with $\mathcal{L}'$ in place of $\mathcal{L}$ and the cultural pool pinned at $Q_{\max}$ throughout, so that $M^* = Q_{\max} + \rho\hat{\vartheta}x^*$ in the interior regime and $M^* = 1$ at the boundary.

G. Crowding costs and the inflated threshold

We introduce a density-dependent cost $\ell(q) = \ell_0 + \gamma q$. The selection gradient on social learning becomes:

$$w_q(q) = \frac{2\mathcal{B}}{1-q} - (\mathcal{L}_0 + \gamma q + \mathcal{B})\,. \tag{85}$$

The critical threshold $q^\dagger$ is the frequency at which this gradient crosses zero. Setting $w_q(q) = 0$ yields the quadratic equation:

$$\gamma q^2 - [\gamma - (\mathcal{L}_0 + \mathcal{B})]q + (\mathcal{B} - \mathcal{L}_0) = 0 \tag{86}$$

Solving for q yields the explicit solution for the invasion threshold under crowding:

$$q^\dagger_{\gamma>0} = \frac{\gamma - (\mathcal{L}_0 + \mathcal{B}) + \sqrt{(\gamma - (\mathcal{L}_0 + \mathcal{B}))^2 + 4\gamma(\mathcal{L}_0 - \mathcal{B})}}{2\gamma}\,. \tag{87}$$

In the limit where crowding costs vanish ($\gamma \to 0$), this expression converges to the standard threshold derived in Eq. (32):

$$\lim_{\gamma\to 0} q^\dagger_{\gamma>0} = \frac{\mathcal{L}_0 - \mathcal{B}}{\mathcal{L}_0 + \mathcal{B}}\,. \tag{88}$$

To verify that the exact solution recovers the linear approximation in the main text (Eq. (36)) when the threshold is small ($q^\dagger \to 0$), we expand the exact solution using a Taylor series. The threshold approaches zero when the difference between the baseline cost and the benefit of bias is small, i.e., $(\mathcal{L}_0 - \mathcal{B}) \to 0$.

Let $\Delta = \mathcal{L}_0 - \mathcal{B}$ and $\Sigma = \mathcal{L}_0 + \mathcal{B}$. Assuming $\Sigma > \gamma$, the exact root is rewritten as:

$$q^\dagger_{\gamma>0} = \frac{\gamma - \Sigma + \sqrt{(\Sigma - \gamma)^2 + 4\gamma\Delta}}{2\gamma}\,. \tag{89}$$

Factoring $(\Sigma - \gamma)^2$ out of the square root yields:

$$q^\dagger_{\gamma>0} = \frac{\gamma - \Sigma + (\Sigma - \gamma)\sqrt{1 + \frac{4\gamma\Delta}{(\Sigma-\gamma)^2}}}{2\gamma}\,. \tag{90}$$

For small Δ, we apply the first-order Taylor expansion $\sqrt{1+x} \approx 1 + \frac{x}{2}$:

$$\sqrt{1 + \frac{4\gamma\Delta}{(\Sigma - \gamma)^2}} \approx 1 + \frac{2\gamma\Delta}{(\Sigma - \gamma)^2}\,. \tag{91}$$

Substituting this approximation back into the numerator:

DRAFT

$$q^{\dagger}_{\gamma>0} \approx \frac{\gamma - \Sigma + (\Sigma-\gamma)\left(1 + \frac{2\gamma\Delta}{(\Sigma-\gamma)^2}\right)}{2\gamma}$$
$$= \frac{\gamma - \Sigma + \Sigma - \gamma + \frac{2\gamma\Delta}{\Sigma-\gamma}}{2\gamma}$$
$$= \frac{\frac{2\gamma\Delta}{\Sigma-\gamma}}{2\gamma} = \frac{\Delta}{\Sigma-\gamma}\,. \quad [92]$$

Replacing Δ and Σ with their original terms recovers the linear approximation:

$$q^{\dagger}_{\gamma>0} \approx \frac{\mathcal{L}_0 - \mathcal{B}}{\mathcal{L}_0 + \mathcal{B} - \gamma}\,. \quad [93]$$

H. Interior equilibrium under crowding costs (q^*)

When social learning is subject to density-dependent crowding costs, the fixed cost is replaced by $\ell(q) = \ell_0 + \gamma q$. If the population successfully crosses the invasion threshold $q^{\dagger}$, the frequency of social learning increases and drives the cultural pool to its accuracy ceiling, M^*.

At this saturation point, the expected payoff of a social learner utilizing optimal bias x^* is:

$$V_{\text{SL}}(q) = 1 - \delta(1-M^*) - c(x^*)^2 - (\ell_0 + \gamma q)\,. \quad [94]$$

The population will reach an interior stable equilibrium q^* when the frequency-dependent payoff of social learning is exactly offset by the competitive cost of crowding, rendering it equal to the constant payoff of individual learning:

$$V_{\text{SL}}(q^*) = V_{\text{IL}} \quad [95]$$

Substituting the explicit payoff function and solving for q^*:

$$q^* = \frac{1 - \delta(1-M^*) - (\ell_0 + c^* + V_{\text{IL}})}{\gamma}\,. \quad [96]$$

The cost of squared optimal bias, defined as $c^* = c(x^*)^2$, depends on whether the bias is an interior solution or bounded at the maximum magnitude. If the optimum is interior ($|x^*| < 1$), then $c^* = c\left(\frac{\delta\rho\hat{\vartheta}}{2c}\right)^2 = \frac{(\delta\rho\hat{\vartheta})^2}{4c} = \mathcal{B}$. If the optimum is bounded ($|x^*| = 1$), then $c^* = c(1)^2 = c$. These are summarized compactly in main text Eq. (39):

$$c^* = \min(\mathcal{B}, c)\,. \quad [97]$$

In the limit where the informational environment at saturation allows social learners to acquire the optimal behavior with perfect reliability ($M^* = 1$), the penalty term $\delta(1-M^*)$ vanishes. This yields the simplified expression for the stable interior rest point:

$$q^*_{\text{sat}} \approx \frac{1 - (\ell_0 + c^* + V_{\text{IL}})}{\gamma}\,. \quad [98]$$

If the net profit in the numerator exceeds the crowding multiplier γ, social learning is bounded only by the population limit, and the strategy evolves to fixation ($q \to 1$).

To determine the stable interior frequency of social learning (q^*) when both density-dependent crowding costs and finite population variance penalties are present, we equate the risk-adjusted fitness of a social learner with that of an individual learner.

In the saturated regime, the probability of a social learner acquiring the optimal behavior is pinned at M^*. The risk-adjusted fitness of a social learner subject to crowding costs $\ell(q) = \ell_0 + \gamma q$ is:

$$W_{\text{SL}}(q) = 1 - \delta(1-M^*) - c(x^*)^2 - \ell_0 - \gamma q - \frac{1}{N_e}\sigma^2_{\text{SL}} \quad [99]$$

where $\sigma^2_{\text{SL}} = \delta^2 M^*(1-M^*)$.

The risk-adjusted fitness of an individual learner remains constant:

$$W_{\text{IL}} = V_{\text{IL}} - \frac{1}{N_e}\sigma^2_{\text{IL}} \quad [100]$$

where $V_{\text{IL}} = 1 - \beta(1-s) - k$ and $\sigma^2_{\text{IL}} = \beta^2 s(1-s)$.

Setting $W_{\text{SL}}(q^*) = W_{\text{IL}}$ and solving for q^* yields:

$$q^* = \frac{1 - \delta(1-M^*) - (\ell_0 + c^* + V_{\text{IL}}) - \frac{1}{N_e}\Delta\sigma^2}{\gamma}\,. \quad [101]$$

We can separate this expression into the infinite-population equilibrium component (q^*_{∞}) and a finite-population modifier. Recall that $\Delta\sigma^2 = \sigma^2_{\text{SL}} - \sigma^2_{\text{IL}}$:

$$q^*_{N_e} = q^*_{\infty} - \frac{\Delta\sigma^2}{\gamma N_e}\,. \quad [102]$$

Clipping this to the valid probability domain $[0,1]$ provides the explicit equilibrium:

$$q^*_{N_e} = \text{clip}_{[0,1]}\left(q^*_{\infty} - \frac{\Delta\sigma^2}{\gamma N_e}\right)\,. \quad [103]$$

This reveals a direct relationship between sampling variance, crowding, and population size. The finite population equilibrium is simply the infinite-population equilibrium shifted by the variance difference scaled by the crowding parameter.

If social learning is the higher-variance strategy ($\Delta\sigma^2 > 0$), the variance penalty reduces its competitiveness, and the population stabilizes at a lower frequency of social learning than would be expected in an infinite population. Conversely, if individual learning has higher variance ($\Delta\sigma^2 < 0$), social learning functions as a variance reduction mechanism, and the finite population size pushes the stable equilibrium to a higher frequency.

I. Finite population threshold dynamics

In finite populations, the condition for social learning to invade ($w_q > 0$) must account for the variance penalty. The risk-adjusted gradient is:

$$w_q(N_e) = (V_{\text{SL}} - V_{\text{IL}}) - \frac{1}{N_e}(\sigma^2_{\text{SL}} - \sigma^2_{\text{IL}})\,. \quad [104]$$

At the invasion boundary ($q \to 0$), the cultural pool is dominated by individual learning, so $Q \approx s$. A mutant social learner will still employ the optimal bias x^*. Let $\Delta_M = \rho\hat{\vartheta}x^*$ represent the informational gain from this bias. The probability of acquiring the optimal behavior is $M^* = s + \Delta_M$.

For the optimally biased social learner:

$$V_{\text{SL}} \approx 1 - \delta(1-M^*) - c(x^*)^2 - \ell \quad [105]$$
$$\sigma^2_{\text{SL}} \approx \delta^2 M^*(1-M^*) \quad [106]$$

For an individual learner:

$$V_{\text{IL}} = 1 - \beta(1-s) - k \quad [107]$$
$$\sigma^2_{\text{IL}} = \beta^2 s(1-s) \quad [108]$$

The effect of finite populations depends on the sign of the variance difference $\Delta\sigma^2 = \sigma^2_{\text{SL}} - \sigma^2_{\text{IL}}$. Substituting the explicit variance terms:

$$\Delta\sigma^2 = \delta^2 M^*(1-M^*) - \beta^2 s(1-s)\,. \quad [109]$$

This difference is determined jointly by the asymmetry in error penalties (δ versus β) and the difference in outcome variance associated with the respective accuracies. Setting $\Delta\sigma^2 = 0$ identifies the condition where finite population size has no net effect on the invasion threshold:

$$\frac{\delta^2}{\beta^2} = \frac{s(1-s)}{M^*(1-M^*)}\,. \quad [110]$$

The left side of the equation represents the ratio of the squared penalties, while the right side represents the ratio of the binomial variances.

- When $\frac{\delta^2}{\beta^2} > \frac{s(1-s)}{M^*(1-M^*)}$, the penalty for social learning errors outweighs the variance reduction provided by the informational gain Δ_M. Social learning functions as a higher-variance strategy, and finite population size strictly increases the threshold $q^{\dagger}$.
- When $\frac{\delta^2}{\beta^2} < \frac{s(1-s)}{M^*(1-M^*)}$, the combination of individual learning error penalties and baseline uncertainty outweighs the risks of social learning. Finite population size effectively decreases the threshold $q^{\dagger}$.

J. Upper bound on individual learning accuracy

There is an absolute upper bound on individual learning accuracy, $\bar{s}$, above which the evolution of social learning is blocked entirely. For social learning to invade, the required break-even success probability $M^{\dagger}$ must not exceed

DRAFT

1. The maximum possible risk-adjusted fitness for a social learner occurs at $M = 1$, where the variance σ^2_{SL} is exactly 0:

$$W_{\mathrm{SL}}(1) = 1 - c(x^*)^2 - \ell \quad [111]$$

If the risk-adjusted fitness of an individual learner exceeds this theoretical maximum, invasion is impossible. Setting $W_{\mathrm{IL}}(\bar{s}) = W_{\mathrm{SL}}(1)$ yields:

$$1 - \beta(1-\bar{s}) - k - \frac{\beta^2 \bar{s}(1-\bar{s})}{N_e} = 1 - c(x^*)^2 - \ell \quad [112]$$

Rearranging terms provides the condition that defines the critical limit $\bar{s}$:

$$\beta(1-\bar{s}) + \frac{\beta^2 \bar{s}(1-\bar{s})}{N_e} = \ell + c(x^*)^2 - k \quad [113]$$

This condition is a quadratic equation in terms of $\bar{s}$. Expanding and grouping by powers of $\bar{s}$ yields:

$$\left(\frac{\beta^2}{N_e}\right)\bar{s}^2 + \left(\beta - \frac{\beta^2}{N_e}\right)\bar{s} + \Big(\ell + c(x^*)^2 - k - \beta\Big) = 0 \quad [114]$$

Applying the quadratic formula provides the explicit analytical limit for individual learning accuracy:

$$\bar{s} = \frac{-\left(\beta - \frac{\beta^2}{N_e}\right) + \sqrt{\left(\beta - \frac{\beta^2}{N_e}\right)^2 - 4\left(\frac{\beta^2}{N_e}\right)\left(\ell + c(x^*)^2 - k - \beta\right)}}{2\left(\frac{\beta^2}{N_e}\right)} \quad [115]$$

For any $s > \bar{s}$, individual learning is highly competitive, and its variance penalty is sufficiently low that the break-even accuracy $M^\dagger$ strictly exceeds 1. Because the social learner cannot achieve a success rate greater than 1, the selection gradient w_q remains negative across all frequencies, and the population is permanently fixed at pure individual learning.

In the limit of an infinite population ($N_e \to \infty$), the sampling variance approaches zero, and the quadratic coefficient $\frac{\beta^2}{N_e}$ vanishes. This reduces the boundary condition to a linear equation:

$$\beta\bar{s} + \ell + c(x^*)^2 - k - \beta = 0 \quad [116]$$

Solving for $\bar{s}$ yields the explicit threshold for infinite populations:

$$\bar{s}_\infty = 1 - \frac{\ell + c(x^*)^2 - k}{\beta} \quad [117]$$

This simplifies to the condition where the expected mean payoff of individual learning exactly equals the absolute maximum payoff of an optimally biased social learner ($V_{\mathrm{IL}}(\bar{s}) = V_{\mathrm{SL}}(1)$).

K. Risk aversion and bias magnitude under expected-utility maximization

The main text derives the inflation of optimal bias x^* under the variance-penalty fitness criterion of (20), where the penalty arises from finite population size. This section provides a parallel derivation under expected-utility maximization with a concave utility function over fitness outcomes, showing that the inflation conclusion does not depend on the specific variance-penalty decomposition: any rational learner who is averse to fitness variance will optimally choose a stronger bias than a risk-neutral learner.

Setup. Consider a social learner with bias x sampling from a pool of quality Q. The learner's fitness outcome is binary: with probability $M(Q,x) = Q + \rho x \vartheta(x)$ she acquires correct behavior and obtains payoff $w_+ = 1 - cx^2 - \ell$, and with probability $1 - M(Q,x)$ she acquires wrong behavior and obtains payoff $w_- = 1 - \delta - cx^2 - \ell$. The two outcomes differ by a constant $w_+ - w_- = \delta$, the mismatch penalty.

A risk-neutral learner maximizes expected fitness $V_{\mathrm{SL}}(Q,x) = M(Q,x)w_+ + (1 - M(Q,x))w_-$. The first-order condition recovers the main text's expression $x^*_{\mathrm{RN}} = \delta\rho\hat{\vartheta}/(2c)$ (main text Eq. (18)) in the interior regime.

A risk-averse learner instead maximizes expected utility $\mathbb{E}[U(W)]$ for some twice-differentiable, strictly increasing, strictly concave U. The second-order Taylor expansion of U around the mean $\bar{w} = V_{\mathrm{SL}}(Q,x)$ gives

$$\mathbb{E}[U(W)] \;\approx\; U(\bar{w}) - \tfrac{1}{2}\,A(\bar{w}) \cdot U'(\bar{w}) \cdot \mathrm{Var}(W), \quad [118]$$

where $A(w) = -U''(w)/U'(w)$ is the Arrow–Pratt coefficient of absolute risk aversion. This approximation is the leading-order correction for any concave U as the variance of W becomes small. The variance of social-learning fitness is the binomial-outcome variance scaled by the mismatch penalty:

$$\mathrm{Var}(W) \;=\; \delta^2 M(Q,x)\Big(1 - M(Q,x)\Big). \quad [119]$$

Optimal bias under risk aversion. Maximizing $V_{\mathrm{SL}}(Q,x) - \frac{1}{2}A(\bar{w})\delta^2 M(1-M)$ over x, treating $A(\bar{w})$ as approximately constant in x (its variation is of higher order in x through the bias cost cx^2, exactly so under constant absolute risk aversion), yields the first-order condition

$$\rho\vartheta\,\delta\left[1 - \tfrac{1}{2}A(\bar{w})\,\delta\,\Big(1 - 2M(Q,x^*)\Big)\right] \;=\; 2cx^*. \quad [120]$$

Solving,

$$x^*_{\mathrm{RA}} \;=\; \frac{\rho\vartheta\delta}{2c}\left[1 - \tfrac{1}{2}A(\bar{w})\,\delta\,\Big(1 - 2M^*\Big)\right] \;=\; x^*_{\mathrm{RN}}\left[1 + \tfrac{1}{2}A(\bar{w})\,\delta\,\Big(2M^* - 1\Big)\right], \quad [121]$$

where $M^* := M(Q, x^*_{\mathrm{RA}})$. Whenever $M^* > 1/2$—which holds for any pool of better-than-random quality ($Q > 1/2$) under any non-negative bias—the bracket exceeds 1, and $|x^*_{\mathrm{RA}}| > |x^*_{\mathrm{RN}}|$. The same conclusion holds for negative bias by symmetry. Optimal bias under risk aversion is strictly larger in magnitude than under risk neutrality. This conclusion applies in the interior regime, where the unconstrained optimum lies in $(-1,1)$. Once the clip at $|x^*| = 1$ binds, the magnitude cannot increase further, and risk aversion has no marginal effect on the bias; the comparative statics in Eq. (121) should be read as the leading-order behavior in the interior.

Comparison with the finite-population result. The bracket in Eq. (121) has the same structure as the risk-adjustment factor in the main text's finite-population result (main text Eq. (41)), with the correspondence $\frac{1}{2}A(\bar{w}) \leftrightarrow 1/N_e$. The two arguments—the finite-population variance penalty and expected-utility maximization with concave U—deliver the same qualitative inflation through different premises and at slightly different rates: the variance-penalty form $V - \sigma^2/N_e$ weighs variance twice as heavily as the second-order Taylor expansion of expected utility, so a finite-population learner of size N_e behaves like a learner with constant absolute risk aversion $A = 2/N_e$. The two-fold difference reflects how the variance-penalty form is derived rather than a substantive disagreement: both predict that more risk-averse learners filter more aggressively, with the inflation factor scaling linearly in the relevant risk-aversion parameter at leading order. The main text's empirical implications about parochial intensification under risk therefore do not depend on the variance-penalty decomposition specifically; any learner who responds to fitness variance—whether through demographic structure, intrinsic risk preference, or both—chooses stronger similarity bias than a risk-neutral counterpart.

Range of validity. The mean–variance form in Eq. (118) is the leading-order expansion of $\mathbb{E}[U(W)]$ in $\mathrm{Var}(W)$. For specific utility families with unbounded marginal utility, the higher-order terms can alter the comparative statics at extreme values of the risk-aversion parameter, and the inflation of $|x^*|$ need not be monotone in A globally; the result Eq. (121) is the leading-order behavior, which is the relevant one for the qualitative claim of the main text.

DRAFT
